# Changing Metabolic Patterns along the Colorectal Adenoma–Carcinoma Sequence

**DOI:** 10.3390/jcm11030721

**Published:** 2022-01-29

**Authors:** Julia Tevini, Sebastian K. Eder, Ursula Huber-Schönauer, David Niederseer, Georg Strebinger, Johanna M. Gostner, Elmar Aigner, Christian Datz, Thomas K. Felder

**Affiliations:** 1 Department of Laboratory Medicine, Paracelsus Medical University, 5020 Salzburg, Austria; j.tevini@salk.at; 2 First Department of Medicine, Paracelsus Medical University, 5020 Salzburg, Austria; sebastian.eder@gmail.com (S.K.E.); e.aigner@salk.at (E.A.); 3 Department of Pediatrics and Adolescent Medicine, St. Anna Children’s Hospital, Medical University of Vienna, 1090 Vienna, Austria; 4 Department of Internal Medicine, Hospital Oberndorf, Teaching Hospital of the Paracelsus Medical University Salzburg, 5110 Oberndorf, Austria; u.huber-schoenauer@salk.at (U.H.-S.); g.strebinger@salk.at (G.S.); 5 Department of Cardiology, University Heart Center Zurich, University Hospital Zurich, University of Zurich, 8091 Zurich, Switzerland; david.niederseer@usz.ch; 6 Institute of Medical Biochemistry, Innsbruck Medical University, 6020 Innsbruck, Austria; johanna.gostner@i-med.ac.at

**Keywords:** colorectal cancer, adenoma, metabolomics, lipid metabolism

## Abstract

Colorectal cancer (CRC) is a major public health burden and one of the leading causes of cancer-related deaths worldwide. Screening programs facilitate early diagnosis and can help to reduce poor outcomes. Serum metabolomics can extract vital molecular information that may increase the sensitivity and specificity of colonoscopy in combination with histopathological examination. The present study identifies serum metabolite patterns of treatment-naïve patients, diagnosed with either advanced adenoma (AA) or CRC in colonoscopy screenings, in the framework of the SAKKOPI (Salzburg Colon Cancer Prevention Initiative) program. We used a targeted flow injection analysis and liquid chromatography-tandem mass spectrometry metabolomics approach (FIA- and LC-MS/MS) to characterise the serum metabolomes of an initial screening cohort and two validation cohorts (in total 66 CRC, 76 AA and 93 controls). The lipidome was significantly perturbed, with a proportion of lipid species being downregulated in CRC patients, as compared to AA and controls. The predominant alterations observed were in the levels of lyso-lipids, glycerophosphocholines and acylcarnitines, but additionally, variations in the quantity of hydroxylated sphingolipids could be detected. Changed amino acid metabolism was restricted mainly to metabolites of the arginine/dimethylarginine/NO synthase pathway. The identified metabolic divergences observed in CRC set the foundation for mechanistic studies to characterise biochemical pathways that become deregulated during progression through the adenoma to carcinoma sequence and highlight the key importance of lipid metabolites. Biomarkers related to these pathways could improve the sensitivity and specificity of diagnosis, as well as the monitoring of therapies.

## 1. Introduction

Colorectal cancer (CRC) is among the three most common forms of malignancy, according to the WHO, and a leading cause of cancer-related deaths on a global level. Despite prevention programs and advancements in therapy, CRC is still the second most common cause of cancer death in Europe, accounting for 12.4% of deaths in 2020 [1,2]. Incidence rates vary geographically, with higher rates in more developed regions and associated with socioeconomic status [3]. Colonoscopy screening effectively reduces CRC-associated mortality, as early stage lesions are removable in time [4].

This study included participants of the SAKKOPI (Salzburg Colon Cancer Prevention Initiative) program [5], who underwent CRC colonoscopy, according to national screening recommendations. Serum metabolomes from participants with diagnosed advanced adenoma (AA) or CRC, as well as from participants without AA or CRC were analysed, providing the rare opportunity to describe the metabolic status in treatment-naïve individuals. Such information is relevant to strengthen the understanding of disease-associated molecular changes and the development of diagnostic markers.

Serum metabolite levels directly reflect the underlying biochemistry and state of cells and tissues. Metabolite concentrations change in response to exogenous triggers (environment, nutrition, lifestyle factors) but importantly, they also reflect endogenous alterations, allowing an early detection of dysregulated and diseased processes. Moreover, changed metabolite profiles may indicate the individual adaptation potential. Pathological alterations and activated immune responses strongly affect the abundance of metabolites in blood. Presumably, the changes in metabolic profiles in a systemic biofluid, such as serum, predominantly reflect a host’s response to the cancer or adenoma, and cellular metabolic changes in the colon may have minor influence. Thus, serum metabolomics can reflect molecular phenotypes associated with healthy or diseased status, with an emphasis on essential changes in immunometabolism that are relevant for disease outcome [6]. Apart from the phenotypic characterisation on a molecular level, metabolomics supports biomarker discovery, essential for genuine personalised medicine.

CRCs mostly arise from epithelial cells and typically include an adenoma to carcinoma sequence [7,8]. The process requires the acquisition of several mutations. Activation of oncogenes and loss of tumour suppressor genes regulate downstream signalling pathways and severe metabolic reprogramming. The crosstalk between oncogenic and metabolic pathways often depends on the protein kinases AKT and c-MYC, and regulates the expression of metabolic enzymes.

Alterations in EGFR/MAPK, Notch, PI3K, TGF-β and Wnt/β-Catenin signalling pathways are frequently involved [9]. As a result, CRC cells adapt glycolysis and the TCA cycle, nucleotide biosynthesis, and the metabolism of lipids and amino acids [10]. These metabolic adaptations occur in adenomas and more advanced malignancies and may even be a prerequisite for the adenoma to carcinoma sequence progression [11,12].

In highly malignant cancers, the metabolic shift is a general finding, irrespective of the origin. The so-called Warburg effect characterises the capability of rapidly growing tumour cells to preserve high rates of glycolysis for adenosine triphosphate (ATP) generation, regardless of oxygen accessibility [13]. On the other hand, recent findings suggest that some cancers might synthesise substantial ATP amounts by oxidative phosphorylation [14]. This finding was termed as reversed Warburg effect, and CRC cells may partially depend on the catabolic pathways of amino acids and lipids, including fatty acid oxidation (FAO) [15].

The deregulated metabolism of amino acids, lipids and aberrant mitochondrial biogenesis regulates growth and proliferation of cancer cells. Glutamine (Gln) is an important nutrient in proliferating tumour cells involved in bioenergetics, defence against oxidative stress and as a precursor for purines and pyrimidines [16]. CRC cells utilise Gln to replenish the TCA cycle in vivo [17]. Gln-derived citrate also provides acetyl-coenzyme A for lipid synthesis, connecting the metabolism of amino acids with lipids. Altered lipid metabolism is a common finding in several cancers and affects lipid biosynthesis and modification, as well as mitochondrial FAO in CRC cells [18,19,20]. Epidemiological studies, including transcriptomic and genomic data, support the crucial role of lipid metabolism in CRC [21,22].

Obesity is a risk factor for CRC and induces the upregulation of lipid metabolic enzymes, such as fatty acid synthase (FASN) and acetyl-coenzyme carboxylase (ACC). These metabolic alterations contribute to CRC progression and metastasis, through activation of oncogenic pathways [23,24,25]. As the metabolism of amino acids and lipids frequently changes in CRC cells, both pathways may provide targets for treatment and management of CRC cases in the future.

The aim of the present study is to characterise serum metabolite profiles of treatment-naïve patients diagnosed with advanced adenoma (AA) or colorectal carcinoma (CRC), compared to controls. We therefore apply quantitative, targeted metabolic profiling of acylcarnitines, amino acids, biogenic amines, glycerophospholipids, and sphingolipids.

## 2. Materials and Methods

### 2.1. Definition of Groups

Included subjects were participants of the Salzburg Colon Cancer Prevention Initiative registry (SAKKOPI). All participants (*n* = 1382 consecutive Caucasians; 702 males (40–76 years) and 680 females (31–88 years)) underwent colonoscopy according to the national CRC screening recommendations at a single centre. The study was conducted according to the guidelines of the Declaration of Helsinki, approved by the local ethics committee (Ethikkommission of the federal state of Salzburg, no. 415-E/1262/2-2010), and informed consent was obtained from all participants. Metabolic characterization included an oral glucose tolerance test (OGTT) as well as measurement of fasting blood glucose and insulin measurement to assess insulin resistance. T2DM was defined as either blood glucose level of ≥200 mg/dL after 2 h following oral glucose tolerance test (OGTT), fasting blood glucose (FBG) ≥ 125 mg/dL or HbA1c ≥ 6.5. Hypertension was defined as a blood pressure (BP) ≥ 130/85 mmHg or previous prescription of any antihypertensive drug.

Colonoscopy screening data from 82 Caucasians were included in the explorative training cohort and allocated to one of the three groups. Control (Control, *n* = 36), advanced adenoma (AA, *n* = 28) with villous or tubulovillous features with a size ≥1 cm or high-grade dysplasia, and colorectal cancer (CRC, *n* = 18) after a combined analysis of macroscopic and histological results [5]. To confirm results from the training cohort, we used two validation cohorts (validation CRC, Control, *n* = 29, CRC, *n* = 48; and validation AA, Control, *n* = 28, AA, *n* = 48) within the SAKKOPI registry, consisting of posterior samples that were not available during the initial training cohort study.

### 2.2. Sample Preparation and Metabolomic Measurements

Serum samples were analysed with the targeted and quantitative AbsoluteIDQ^®^ p180 kit (Biocrates life science AG, Innsbruck, Austria) by mass spectrometry according to the manufacturer’s guidelines. The assay allows the quantification of up to 188 metabolites from five analytical groups: acylcarnitines, amino acids, biogenic amines, glycerophospholipids and sphingolipids.

All samples were stored at −70 °C until measurements. Samples were thawed, vortexed and centrifuged at 4 °C for 10 min at 14,000× *g*. Next, 10 µL of the supernatants were transferred onto the spots of the 96-well kit plate. The samples were dried at room temperature under a gentle stream of nitrogen. After drying the filter spots, amino acid derivatisation ensued with 5% phenyl isothiocyanate reagent (*v*/*v*, PITC). Filters were dried again and metabolites as well as the internal standard were extracted by adding 5 mM ammonium acetate in methanol. After centrifugation of the filter plate for 2 min at 500× *g*, the flow-through extracts were diluted with either HPLC water or MS-running solvent. We used an Agilent 1200 Series HPLC coupled to an API 4000 triple quadrupole mass spectrometer (ABSciex, Darmstadt, Germany) controlled by the software Analyst 1.6. Calibrators, quality controls and samples were analysed by an LC-MS/MS method in positive electrospray ionization mode (amino acids, biogenic amines) followed by FIA-MS/MS injections (acylcarnitines, lipids, hexose) in positive and negative mode. Metabolites were quantified by multiple reaction monitoring (MRM) detection in reference to stable isotope-labelled and chemically homologous internal standards. We used the integral MetIDQ software package for data assessment, evaluation and quantification of metabolite concentrations.

### 2.3. Statistical Analyses

All statistical calculations were performed using SPSS Statistics (Version 24.0.0.1, IBM SPSS Statistics for Windows, Released 2018, IBM Corporation, Armonk, NY, USA). Analyses included 228 variables consisting of quantified metabolites as well as predefined ratios and sums, e.g., sum of all glycerophosphocholine species (total PC) or the kynurenine to tryptophan ratio (Kyn/Trp), respectively. We analysed differences between groups (Control, AA, CRC) regarding parametric variables and conducted multivariate analysis of variance (MANOVA) applying Benjamini–Hochberg or Tamhane (FDR) correction for multiple testing. Non-parametric distributed variables were analysed using Kruskal–Wallis test.

Exploratory statistical analyses such as sparse partial least square discriminant analysis (sPLS–DA), heat maps, pattern analyses and receiver operating characteristic (ROC) curve-based biomarker analyses were performed with the online statistical analysis tool MetaboAnalyst 4.0 (www.metaboanalyst.ca, accessed on 10 June 2020). We skipped missing value estimation, data normalization, data transformation or data scaling for this purpose. For heat maps, options such as autoscale by features for standardisation, Euclidean distance measure and Ward clustering were chosen. For pattern analyses, Spearman’s rank correlation coefficient as a distance measure was used.

Statistical analyses of the two validation cohorts included the same variables as in the training cohort. Since only two groups were analysed within each validation cohort, means of these groups were compared by either an unpaired *t*-test or a Mann–Whitney U test depending on data distribution. A two-sided *p*-value of <0.05 and a z- or *t*-value ≥ 1.96 were considered to indicate statistical significance.

## 3. Results

### 3.1. Clinical Characteristics

To identify group differences in basic demographic and clinical features of the training cohort, we performed descriptive statistics, ANOVA and corrected for multiple testing (Table 1). All groups were similar in sex distribution and BMI, but the control group was younger than both the adenoma and carcinoma group. Liver parameters aspartate aminotransferase (AST), alanine aminotransferase (ALT) and γ-glutamyl transferase (GGT) and concentrations of serum triglycerides were similar between all groups. Control subjects had normal ultrasound examinations with a homogenous echogenicity compared to renal parenchyma, while 29% of AA and 22% of CRC had unequivocal evidence of fatty liver. In contrast to the AA and CRC group, controls had no diabetes and lower fasting glucose (FG) values. HDL–C decreased from the controls to the adenoma and to the carcinoma group. Features of the metabolic syndrome (MetS) differed significantly between the CRC and the control group. CRP levels were different between all groups and increased from control via adenoma to the carcinoma group. The observed group differences demand a more extensive analysis to decipher underlying metabolic events.

### 3.2. Metabolic Profiling

**Figure 1:**
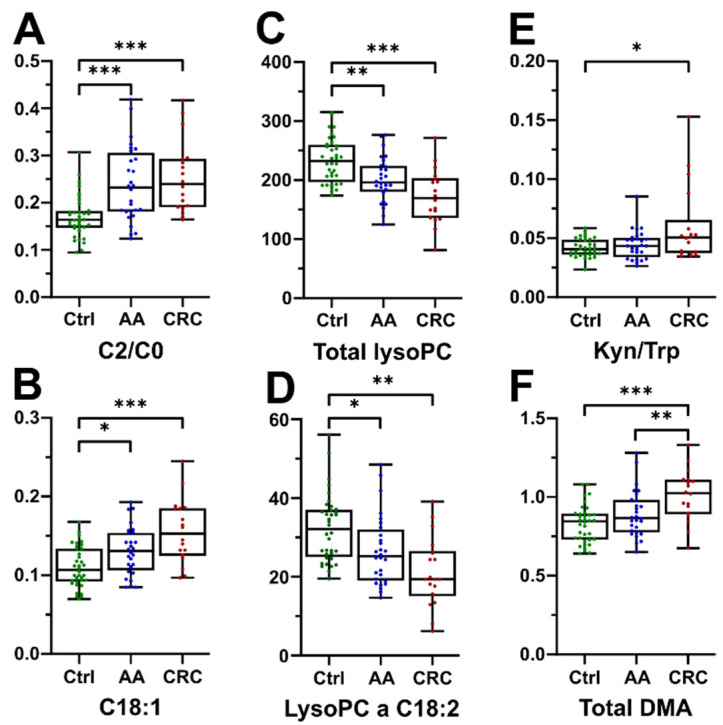
Selected statistically significant parameters. (**A**) Ratio of acetylcarnitine to free carnitine and (**B**) octadecenoylcarnitine; (**C**) sum of all lysoPC species and (**D**) the specific lysoPC a C18:2; (**E**) ratio of kynurenine to tryptophan; (**F**) total dimethylarginine; data are expressed in µM (except for ratios); * indicates *p*-values < 0.05, ** *p*-values < 0.01, *** *p*-values < 0.001.

**Figure 2:**
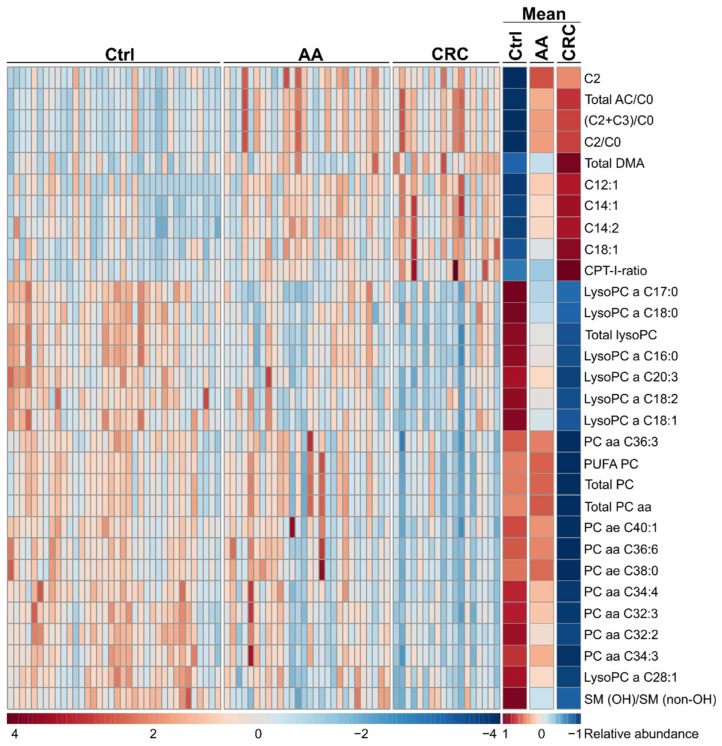
Heatmap of top 30 features derived from ANOVA for individual patients (**left**) and group mean averages (**right**) presented without clustering. AC, acylcarnitine; DMA, dimethylarginine; lysoPC, monoacyl-glycerophosphocholine; PC aa, diacyl-glycerophosphocholine; PC ae, alkyl-acyl-glycerophosphocholine; PUFA, poly-unsaturated fatty acid; SM, sphingomyelin; abundance of each of the metabolites is presented by colour ranging from low (blue) over average (white) to high (red); adapted from MetaboAnalyst 4.0.

We used targeted metabolomics to identify the differences of acylcarnitines, amino acids, biogenic amines, and lipids between the groups. The results for all single features and derived variables (ratios and sums) are presented in Supplementary Table S1. Groups mainly differed by lipids, including monoacyl-glycerophosphocholines (lysoPC), acylcarnitines (AC), diacyl-glycerophosphocholines (PC aa), alkyl-acyl-glycerophosphocholines (PC ae) and derived parameters. We observed less pronounced differences in amino acids and catabolites thereof between the groups (Table 2, Figure 1). The top 30 metabolites, ratios and sums are presented by a hierarchical clustering heatmap for intuitive visualization in Figure 2.

Many ACs were significantly more abundant in AA (12 of 27 ACs) and CRC patients (14 of 27 ACs) compared to controls, whereas AA and CRC groups did not differ significantly (Figure 1A,B, Figure 2 and Table 2). In line with this finding, the CPT–I-ratio (ratio of C16 and C18 long chain acylcarnitines to free carnitine) was significantly different between all groups. The CPT–I-ratio is a suggested proxy measure of ß-oxidation. Therefore, our results indicate increased ß-oxidation in the control, to adenoma, to CRC sequence.

Among the glycerophospholipid class, levels of several lysoPC species were low in the CRC group, as compared to controls (Figure 1C,D). This was not true for arachidonic acid containing lysoPC a C20:4 levels, which were similar in all three groups. In addition, many ester-bound phosphatidylcholine species (diacyl-glycerophosphocholines, PC aa) were least abundant in the CRC group. CRC patients also presented the lowest concentrations of many (21 of 38 species) ether-bound plasmanylcholines (alkyl-acyl-glycerophosphocholines, PC ae), which were significantly reduced compared to controls. In addition, levels of some sphingolipids were decreased in CRC patients. Five of fifteen sphingomyelins were reduced in the CRC group and included mostly hydroxylated forms.

The increase of ACs in the AA and CRC group and the concomitant reduction of glycerophospholipids and sphingomyelins in CRC point to alterations in lipid metabolism. Our results indicate altered biosynthesis, metabolism or degradation of hydroxylated sphingomyelins by lysosomal and peroxisomal pathways during the adenoma to carcinoma sequence.

We observed a lower number of statistically different analytes between the groups concerning amino acids. Controls had higher glycine concentrations than AA patients and histidine was significantly lower in CRC than in control subjects. CRC patients showed a trend for decreased Gln levels compared to controls, but levels were similar as in AA patients. The kynurenine to tryptophan ratio (Kyn/Trp, Figure 1E) was significantly higher in CRC compared to controls, but similar to the AA group, while isolated kynurenine (Kyn) and tryptophan (Trp) concentrations were similar in all groups. AA subjects had the highest ornithine levels, which were statistically different only to the control group. CRC patients had significantly decreased citrulline to arginine ratios (Cit/Arg), a marker for nitric oxide synthase (NOS) activity. In contrast, the ornithine to arginine ratio (Orn/Arg), indicative for arginase activity, was lowest in CRC patients. Total dimethylarginine (total DMA, Figure 1F) concentrations were significantly higher in the CRC group, compared to both AA and control groups. Serum levels of the oxidative stress biomarker methionine sulfoxide (MetSO) were lowest in the control group.

Overall, amino acid alterations were less pronounced than those of lipids among the adenoma to carcinoma sequence. Modifications included Trp breakdown, NOS activity and levels of total DMA. As total DMA increased from control to adenoma to carcinoma and inhibits NO synthesis, our data may indicate the existence of possible biomarkers along this pathway.

### 3.3. Clustering of Groups

**Figure 3:**
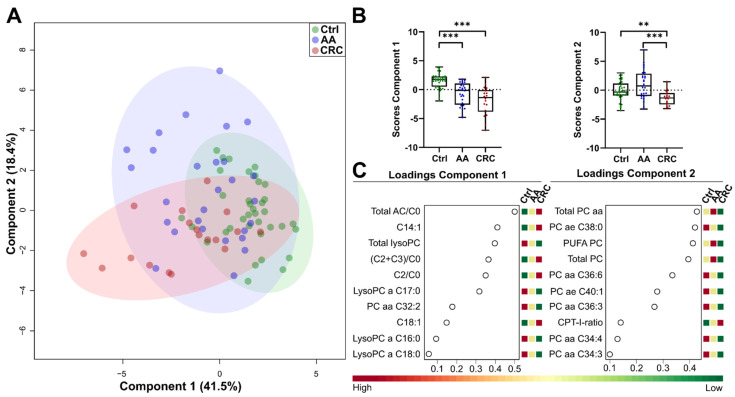
Sparse partial least discriminant analysis (sPLS-DA) between Control (green), AA (blue) and CRC (red) samples. (**A**) Score plot including Component 1 and Component 2, circles indicate 95% CI; (**B**) separation of Control, AA and CRC by the first and second component; (**C**) loading plot of top 10 features used by Component 1 (**left**) and Component 2 (**right**); AC, acylcarnitine; LysoPC, monoacyl-glycerophosphocholine; PC aa, diacyl-glycerophosphocholine; PC ae, alkyl-acyl-glycerophosphocholine; PUFA, poly-unsaturated fatty acid; adapted from MetaboAnalyst 4.0; ** indicates *p*-values < 0.01, *** *p*-values < 0.001.

Next, we used sparse partial least squares discriminant analysis (sPLS–DA), with the top 30 features from ANOVA, to promote the identification of features relevant to the adenoma to carcinoma sequence. The Kaiser–Meyer–Olkin measure was 0.725, indicative for appropriate analysis, and Bartlett’s test of sphericity was significant (*p* < 0.001), illustrating sufficient correlations between the items for further analysis. Values of the Kaiser’s criteria and visual examination of the scree plot legitimated the analysis with two fixed components, accounting for 41.5% on Component 1 and 18.4% on Component 2, respectively. As such, 59.9% of total variance allowed distinguishing between the groups (Figure 3A). The score values of the two components significantly separated the groups (Figure 3B). The most important metabolites for Component 1 included lysoPCs, ACs with derived parameters and PC aa C32:2. Metabolites from Component 2 mainly consisted of PC aa and PC ae molecular species (Figure 3C). Thus, data reduction by sPLS–DA yielded a moderate clustering of groups, but strongly indicated differences, mainly in the lipid profiles of control, AA and CRC patients.

### 3.4. Correlation, Pattern Discovery and ROC Analyses

**Figure 4:**
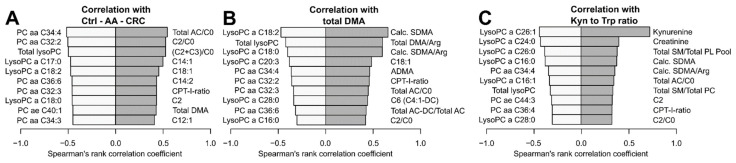
Pattern analyses showing negative (left bar, light grey) and positive (right bar, dark grey) correlation based on Spearman’s rank correlation coefficient. (**A**) Metabolites correlating within the control to adenoma to carcinoma sequence; (**B**) metabolites correlating with the total amount of DMA; (**C**) metabolites correlating with the Kynurenine to Tryptophan ratio; adapted from MetaboAnalyst 4.0.

We analysed metabolites with pattern-specific concentration differences between the groups. First, we focused on identifying metabolites with increased concentration along the control to adenoma to carcinoma sequence (Figure 4A). The acetylcarnitine to free carnitine ratio (C2/C0) showed a strong positive correlation (0.544). We observed moderate correlation for other ACs and total DMA. All positive and negative correlation coefficients are presented in Supplementary Tables S2–S4. The strongest negative correlation within this sequence was observed for the glycerophospholipid PC aa C34:4 (−0.516). Other negatively correlated metabolites included PCs and lysoPCs. Overall, our results indicate modifications of lipid metabolism along the CRC adenoma to carcinoma sequence.

Our previous results indicated a possible role of DMA in the adenoma to carcinoma sequence. DMA metabolism connects to the nitric oxide (NO) pathway, and associates with systemic immune activation. Therefore, we aimed to identify patterns associated with the total amount of DMA (Figure 4B). Total DMA consists of two analogues, symmetric dimethylarginine (SDMA) and asymmetric dimethylarginine (ADMA). Both metabolites and derived parameters, as well as ACs, correlated positively. Features with negative correlation were similar to those found in the analysis of the disease sequence and contained lipids. The DMA results from above may indicate systemic immune activation. Therefore, we assessed patterns associated with the Kyn/Trp ratio, a well-known parameter of immune activation in CRC (Figure 4C) [26]. As expected, Kyn levels correlated strongly (0.717), whereas creatinine, derived DMA and some AC parameters were only moderately correlated. Mainly unsaturated or mono-unsaturated lysoPCs and the total amount of lyso-lipids were negatively correlated. The findings confirm altered lipid metabolism along the control to adenoma to carcinoma sequence.

**Figure 5:**
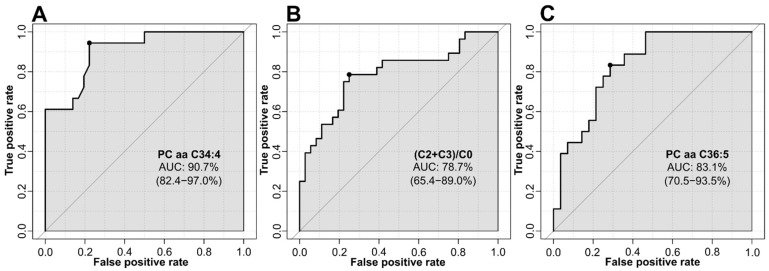
Univariate ROC analyses based on area under the curve (AUC) values and 95% CIs. (**A**) ROC analysis between control and CRC; (**B**) ROC analysis between control and AA; (**C**) ROC analysis between AA and CRC; adapted from MetaboAnalyst 4.0.

Next, we identified possible biomarkers discriminating between the metabolic groups, and performed univariate receiver operating characteristic (ROC) analyses. The comparison of the control and CRC group identified PC aa C34:4, with an area under the curve (AUC) of 90.7%, as the best predictor (Figure 5A). The discriminating power between control and AA subjects for the ratio of short chain ACs to free carnitine ((C2 + C3)/C0) reached an AUC of 78.7% (Figure 5B). On the other hand, PC aa C36:5 discriminated AA and CRC subjects, with an AUC of 83.1% (Figure 5C). Results of top 10 predictors for all possible group comparisons are summarised in Supplementary Table S5. Therefore, univariate ROC curve analysis revealed that lipids had the highest power for discrimination of groups.

### 3.5. Validation Cohorts

To validate our findings, we analysed serum metabolite profiles in two separate validation cohorts (validation CRC and validation AA). We assessed differences in demographic and laboratory characteristics between the training cohort and the validation cohorts (Table 3).

Groups of both validation cohorts showed minor differences in terms of age, sex distribution and parameter of lipid metabolism compared to the groups of the training cohort. The percentage of subjects within all groups of both validation cohorts with fatty liver was higher in the validation cohorts compared to the training cohort. The BMI and GGT of the control group in the validation AA cohort was significantly higher than in the training cohort. Levels of triglyceride and HDL–C differed between CRC subjects of the training and validation cohorts. Despite some differences, the validation cohorts were mainly similar in major characteristics.

We performed identical analytical mass spectrometric and statistical analyses, as for the training cohort. Results of sPLS–DA are presented in Supplemental Figure S1. Results of metabolic features are summarised in Supplementary Table S6 for both validation cohorts, respectively.

In the validation AA cohort, controls differed from AA subjects, mainly in lipids, amino acids and derived parameters. In contrast to our findings from the training cohort, ACs remained mostly unchanged. Among amino acids, Cit/Arg ratio (indicator for NOS activity) and the mainly proteinogenic amino acids aspartate, glycine and serine were affected. Altered lipids tended to be higher in AA subjects compared to controls (Table 4, Supplementary Table S6). The findings in amino acids were unique to the validation cohort. Of note, absolute values of Kyn and Trp differed between all control subjects of the study. As such, Trp concentrations were lower and Kyn concentrations were higher in both validation cohorts compared to the training cohort. Neither DMA nor Trp, Kyn or the Kyn/Trp ratio was significantly different when comparing AA or CRC with their respective control groups.

For the validation CRC cohort, 67 metabolites and derived parameters were significantly different between control and CRC subjects. In line with results from the training cohort, we found less pronounced differences in amino acid metabolism. Glycine (*p* = 0.028) and histidine (*p* = 0.034) decreased and isoleucine (*p* = 0.003) increased in the CRC group. The reduction of glycine and elevation of isoleucine did not reach statistical significance in the training cohort. On the other hand, our histidine findings confirm the training cohort results. Alterations of lipid metabolism were more evident, and many lipids were present at a lower concentration in the CRC group (Table 4, Supplementary Table S6). On the other hand, some ACs were more abundant in the CRC group. Both findings confirm our results from the training cohort. Furthermore, lyso-lipids with very long chain fatty acids (≥22 carbon atoms) and some lysoPCs with long chain fatty acids (lysoPC a C17:x and lysoPC a C18:x) were among the significantly reduced metabolites. CRC patients had significantly lower levels of PC species (PC ae C36:2 and PC aa C32:3) and reduced hydroxy sphingomyelins. This finding confirms and strengthens our initial results from the training cohort and points to the possible role of sphingolipid hydroxylation in the AA to CRC sequence.

**Figure 6:**
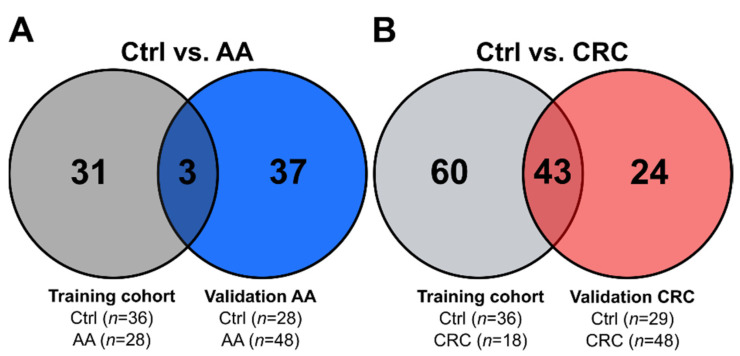
Venn diagrams indicating overlap between significantly different features. (**A**) Overlap between the training cohort and validation CRC; (**B**) overlap between the training cohort and validation AA.

We then analysed the overlap between the training and validation cohorts. We used the top 20 analytes that drive separation in the sPLS–DA training set model to test the validation groups (Supplementary Figure S1). PLS–DA indicated a robust model for the CRC validation cohort, as derived from permutation testing (*n* = 1000; *p* < 0.01). PLS–DA and permutation testing for the AA validation cohort only reached borderline significance (*n* = 1000; *p* = 0.07), indicative for a less robust model. The results for two validated metabolites of the training cohort and both validation cohorts are presented in Supplementary Figure S2. For AA and control subjects, only a moderate overlap between results of the training cohort and the validation AA cohort was observed. Three confirmed metabolites (glycine, the MetSO/Met ratio and SM C18:1) indicate that the majority of regulated metabolites differed to a greater extent than in CRC patients (Figure 6A). Our results may also point to more than subtle underlying differences in the control groups or the effects of repeated freeze–thaw cycles on the validation cohort samples. The overlap of results between the training and validation CRC group was much stronger. Comparison revealed 43 overlapping metabolite features (Figure 6B). Despite the intrinsic heterogeneity in CRC and diverse sample quality, many findings from the training cohort were validated. We confirmed mainly altered lyso-lipids, PC species and ACs between control and CRC subjects.

## 4. Discussion

Alterations of serum metabolic profiles reflect the host response to pathological changes, thus, strengthening the effects of cellular metabolic changes in the colon. Serum metabolome changes can identify the very early stages of disease, e.g., with occult metastasis, and support risk stratification, e.g., by detecting residual metastasis [27,28]. Moreover, the site of the disease may have an impact [27].

A special feature of the present study is that the serum metabolome profiles of a treatment-naïve, random sampling, cross-sectional cohort was available. Patients were diagnosed through the screening program with AA or CRC.

Baseline characteristics provide a hint towards increased inflammation in the control to adenoma to carcinoma sequence. This is reflected by increasing CRP concentrations, probably triggered by metabolic reprogramming. In line with this, the accumulation of MetS features indicates metabolic alterations being more prevalent in the carcinoma compared to the adenoma group. The role of lipid metabolism along the adenoma to carcinoma sequence is apparent. It includes declining HDL-C concentrations and a higher prevalence of fatty liver, increasing from controls to adenoma to carcinoma patients. Findings of the targeted metabolomics approach further confirm these observations, as metabolism of lipids, specifically acylcarnitines (ACs), glycerophosphocholines (PC aa, PC ae), and to a lesser extent, amino acids and catabolites, differed between the groups.

Levels of ACs increased in the AA and CRC groups. Furthermore, the elevated CPT-I-ratio (a proxy measure of ß-oxidation) increased along the adenoma to carcinoma sequence. Increased β-oxidation is a hallmark of chemo- and radiotherapy resistant tumour cells [29]. Our findings may provide preliminary evidence for a reversed Warburg effect in treatment-naïve CRC. In postoperative material, CRC regions had higher rates of oxidative phosphorylation compared to surrounding and healthy colon tissue cells [15]. It was speculated that human CRC is less of a hypoxic tumour, with higher respiration rates. Furthermore, CRC cells have the ability to modulate the energy metabolism of neighbouring cells [15]. The proto-oncogene *MYC* is the proposed master regulator of colorectal tumour metabolism, including a reversed Warburg effect [12]. The protein kinase c-MYC regulates the expression of many enzymes of lipid and amino acid metabolism. Reprogramming and enhancement of lipid metabolism in tumorigenesis is common within certain cancer entities and often includes de novo lipogenesis and uptake from systemic circulation [30].

Alterations in the metabolism of glycerophospholipids are known to contribute to oncogenesis, as well as tumour progression [31], and this this became evident in the SAKKOPI cohort, too. Total lysoPC, as well as some specific lysoPCs, were significantly lower in the AA and CRC group compared to control subjects. Identical lipids were found by another study, as Zhao et al. reported significantly reduced plasma levels of total lysoPC, lysoPC C18:1 and lysoPC C18:2 in CRC [32]. Other CRC plasma lipidomics results confirmed our findings, but also emphasised that even lipid species belonging to the same lipid class may follow different trends in an experimental setting [20]. Reduced levels of lysoPCs may originate from decreased formation and/or increased remodelling activities. Remodelling by liver secreted lecithin:cholesterol acyltransferase (LCAT) transfers fatty acids from PCs to cholesterol-forming lysoPCs. LCAT is repeatedly described as a prognostic biomarker for the detection of HCC and for epithelial ovarian cancer [33,34,35,36,37].

Food intake affects the metabolism of subjects as well as of their gut microbiomes. Serum lysoPC a C17:0 levels were significantly reduced in CRC patients in the presented study. The lipid contains one chain of margaric acid, which is mainly derived from butter, milk and ruminant fat. Interestingly, lysoPC C17:0 was reported to discriminate patients with hepatocellular carcinoma (HCC) from controls [38]. Different serum lysoPC 17:0 levels were also observed in CRC patients and cirrhotic controls [39]. Unfortunately, no data concerning food intake were available.

Lipid modifications, such as desaturation, elongation or hydroxylation, determine the fate of cancer cells [40]. LysoPCs with poly-unsaturated fatty acids (lysoPC a C18:2 and lysoPC a C20:4) were reduced in the CRC patients of our study. The observation may mirror the high demand of proliferating cancer cells for PUFAs and uptake from systemic circulation, for re-acylation and further use as cell membrane components. In line with this, decreased levels in n-3 and n-6 serum PUFAs in CRC were reported earlier [41]. In contrast, the intra-tumour concentrations of PUFAs in CRC patients were higher.

We also observed the changed metabolism of sphingolipids in our study. These complex and structurally different lipid species are involved in many cellular functions, such as membrane components or the regulation of apoptosis and inflammation [42,43,44]. We detected decreased serum levels of distinct SM species in CRC, while AA subjects showed slightly increased or unchanged levels compared to control subjects. The altered ratio of hydroxylated to non-hydroxylated sphingomyelins in AA and CRC patients may point to a possible role of sphingolipid hydroxylation in CRC. The general reduction of SMs in CRC could originate from the altered expression and activity of enzymes (i.e., sphingomelinases, SMase) regulating their metabolism. SMase activity in the colonic mucosa of mice also responds to different types of diet [45]. Additional information on altered alkaline SMase protein expression within different histological stages of colorectal adenomas underscores the crucial role of sphingolipids [46]. If such local changes in enzymatic activity have an impact on the metabolic serum profile solely, this needs further clarification.

Alterations in lipid metabolism may also affect immune cells in CRC. The accumulation of lipids in macrophages creates dysfunctional and pro-inflammatory states in many diseases [47,48,49]. The immune suppressive phenotype of tumour-associated macrophages in CRC may also respond to changes in lipid metabolism [50].

The changed metabolism of circulating immune cells may be a major contributor to altered lipid patterns in serum. There are several biochemical links between the metabolism of lipids and other metabolites, e.g., amino acids and derivatives. For example, up-regulation of the Trp catabolizing enzyme indoleamine 2,3-dioxygenase 1 (IDO-1) in circulating immune cells was reported for several cancer entities, including colorectal, lung and breast cancer, leading to increased serum concentrations of Kyn [51,52]. Murine CD4+ T cells, exposed to Kyn, undergo increased β-oxidation and deplete fatty acids [53]. Kyn and downstream products contribute to the metabolic reprogramming of immune cells via aryl hydrocarbon receptor (AhR) signalling, inhibit glycolysis and increase lipid oxidation [54]. The major driver of inflammation-associated IDO-1 activity is IFN-γ [55]. Metabolites in the Kyn pathway can suppress T cell proliferation, drive T cell apoptosis and induction of regulatory T cells. Serum Kyn/Trp ratios in CRC patients were significantly higher compared to control patients [26]. These findings are in accordance with our training cohort results, with increased serum Kyn/Trp ratios in the CRC group compared to controls, while levels between control and AA subjects were similar. Local Trp catabolism in the tumour creates a tumour-specific microenvironment for immune escape [56]. Expression of IDO-1 in CRC correlated with reduced infiltration by CD3+ T cells and increased rates of hepatic metastases [57]. Colon cancer cells also increase their uptake of the essential amino acid Trp by Myc dependent upregulation of transporters [58].

Chronic inflammation is a risk factor for CRC, as observed in patients with long-standing inflammatory bowel disease, a precancerous condition [59,60,61]. Induced by inflammatory cytokines IFN-γ or TNF-α, epithelial cells in the colon become major IDO-1 expressing cells, causing DNA damage by oxidative stress and the innate immune system [62,63,64]. Recently, IDO-1 expressing Paneth cells in the stem cell niche of intestinal crypts and tumours were described, which promoted immune escape of CRC [65]. IDO-1 expression therefore links inflammation with lipid and amino acid metabolism.

Apart from alterations in Trp metabolism, other differences in amino acid metabolism were minor. Reduced Gln levels were reported in CRC earlier [66]. Interestingly, Gln levels were not different between the groups and only tended to be lower in AA and CRC. Myc also regulates the uptake and degradation of Gln [67]. The reprogramming to Gln catabolism provides citrate and acetyl-coenzyme A, for further lipid synthesis, and intermediates to preserve TCA cycle activity, as well as macromolecular precursors [16].

One explanation for our Gln findings may be associated with the fact that our data also revealed a possible shift of arginine metabolism towards total DMA (i.e., sum of ADMA and SDMA) formation, at the expense of NO metabolism. The Cit/Arg ratio (a proxy for arginase activity) was decreased in CRC compared to controls and points to low NOS activity. This may contribute to decreased metabolism of Gln, polyamine and proline [68]. Increased ADMA and SDMA levels also occur in haematological malignancies [69]. Dimethylamines are methylated degradation products of proteins [70]. The increase in total DMA levels in the CRC group in this dataset is in accordance with Li et al., who reported higher ADMA serum levels in CRC patients [71]. SDMA is removed by renal excretion, thus ADMA is the major contributor for endogenous DMA and is formed via arginine methylation by arginine N-methyltransferase (PRMT). ADMA is an endogenous inhibitor of NOS and the L-Arg/NO pathway is altered in CRC [70,72]. NO has concentration-dependent diverse pro- and anti-tumour effects in cancers. Endogenous NO levels may promote colon neoplasms, as NO affects many CRC signalling pathways associated with inflammation, cancer initiation and metastasis [73]. The decrease in Cit/Arg ratios in our CRC group may indicate a dysregulation of NOS, followed by a lower recycling rate of Cit to argininosuccinate by ASS1 (argininosuccinate synthase 1) and ASL (argininosuccinate lyase), back to Arg [74].

Metabolic alterations along the Arg/ADMA pathway may provide useful biomarkers for CRC tumour progression in future studies. Interestingly, Arg is the most consumed amino acid in the inner necrotic core of tumour mass [75]. There is also some evidence that elevated ADMA levels influence the depth of tumour invasion and poor clinical outcomes in gastric cancer samples [76]. Of note, there is crosstalk between ADMA and lipid metabolism. ADMA treatment of macrophages impaired lipid metabolism and increased NADPH oxidase (NOX) activity and ROS production. Furthermore, ADMA upregulated the expression of pro-inflammatory mediators in macrophages [77,78]. In vivo administration of ADMA worsened aortic inflammation, impaired cholesterol metabolism and promoted atherosclerosis in apoE^−/−^ mice. In line with this, ADMA was suggested to contribute to the regulation of macrophage lipid metabolism, during their transformation to foam cells [79].

### Limitations of the Study

Some common technical drawbacks need consideration. In general, investigations on the serum metabolome rarely provide clear information on the origin of the metabolites and may not exactly reflect the carcinogenic development. Furthermore, the used targeted kit encounters the problem of potential isobaric and isomeric lipid species, as well as the intrinsic limitation of analyte selection.

In addition, this work suffers from potential study-specific limitations. Participants were enrolled in a screening program. Therefore, the variable and early sampling time point could contribute to the weak differences observed for established markers and account for the moderate overlap of cohorts. As an advantage, the samples were taken before any treatment for AA or CRC was set in place. Unfortunately, we do not have comprehensive data on pre-screening medication, food intake or staging information of the participants for the study. Certain medications can affect the blood levels of distinct metabolites, as observed for ADMA and SDMA in patients [80]. Notably, the control population of the training cohort was somewhat younger. Age-related changes of physiological metabolite concentrations are known for some metabolites, e.g., for Trp breakdown along the kynurenine axis, which increases with age, most likely due to low level inflammatory processes [81]. The comparison of absolute concentrations of Kyn and Trp (a more stable metabolite) between all control subjects demonstrated differences between our cohorts. Control subjects of both validation cohorts (validation CRC and validation AA) had reduced Trp but higher Kyn levels compared to the training cohort. The somehow distorted Kyn and Trp serum levels indicate a higher between-cohort variation.

Though a subgroup analysis would be interesting, to better understand the impact of routine laboratory parameter differences or age, Control, AA and CRC groups of the different cohorts were too small for a conclusive subgroup analysis. In this work, cohorts are defined by time of participation in the study and sample availability. Participating subjects represent a random cross-section and reflect real life random sampling. No preselection intrinsically causes higher sample heterogeneity but may lead to more stable markers.

Another limitation of our study is the lack of a truly independent population for validation. Therefore, samples of the validation cohorts consisted of posterior samples from the SAKKOPI registry that were not available to us during the enrolment of the initial study. As mentioned above, several freeze–thaw cycles cannot be fully excluded for the validation cohorts, while the samples of the training cohort were thawed only once for this metabolic study. Results from analytes with reduced stability during the preanalytical phase are more likely impaired. Ex vivo plasma concentrations showed a considerable plasma Arg decrease in short-term stored samples at room temperature compared to storage on ice, while plasma Trp concentrations were stable at both conditions [82]. Knowledge about the analytical stability of lipid metabolites is much more limited; however, elevated levels of fatty acids or glycerol metabolites are potential consequences of thawed samples [83]. Results from our study directly point to sample heterogeneity in terms of quality. Levels of the oxidative stress biomarker MetSO were significantly higher in all samples of the validation cohorts.

Despite all limitations, this study provides the unique opportunity to discover yet unknown patterns, such as lipid profile characteristics, which might be otherwise masked by ongoing treatments. Hence, untargeted methods for future studies and/or validation, with well-defined large cohorts, are still necessary to understand the different metabolic alterations in the adenoma to carcinoma sequence in CRC.

## 5. Conclusions

CRC accounts for many cancer-related deaths due to late detection and suboptimal risk stratification. Alterations in oncogene and tumour suppressor gene expression, as well as host responses, induce pronounced metabolic reprogramming. Changes of the serum metabolome may be indicative for the CRC adenoma to carcinoma sequence and triggered immune responses.

This study investigated sera from a treatment-naïve random sampling cross-sectional cohort. Lipid patterns, and to a lesser extent amino acids, were identified to significantly differ between Control, AA and CRC. A number of lysoPC and ester-bound phosphatidylcholine species, as well as ether-bound plasmanylcholines and sphingolipid species were lower in CRC compared to Control. Further changes concerned the Arg/ADMA axis and ADMA/NOS interaction, as well as sphingolipid hydroxylation. The role of alterations in the metabolism of glycerophospholipids and acylcarnitines, the preliminary evidence for increased β-oxidation and hints for a potential reversed Warburg effect in CRC await further clarification.

In summary, mainly serum lipid and, to a lesser extent, amino acid profiles changed significantly with the progression of the adenoma to CRC sequence. The roles of lipids in the pathogenesis of CRC merit further investigation, as lipid patterns bear a great potential as clinically useful markers for personalised medicine. The combination of established and novel metabolites may define new standards and strategies in the early diagnosis and treatment of CRC, which may foster the generation of novel therapeutic regimens that improve outcomes, in addition or as alternatives to conventional therapy.

## Figures and Tables

**Table 1:** Baseline characteristics of the training cohort.

	Control (*n* = 36)	AA (*n* = 28)	CRC (*n* = 18)	Post Hoc *p*-Value		
				Control vs. AA	Control vs. CRC	AA vs. CRC
Age (years)	53 ± 8	60 ± 10	67 ± 12	**<0.001**	**0.001**	0.271
Gender (f/m)	18/18	14/14	7/11	1.000	0.444	0.465
BMI (kg/m^2^)	25.7 ± 2.8	26.6 ± 4.7	26.2 ± 3.6	0.892	0.956	1.000
Waist circumference (cm)	95 ± 12	97 ± 14	99 ± 14	0.645	0.286	0.582
Hip circumference (cm)	98 ± 11	103 ± 10	100 ± 10	0.723	0.940	0.541
Waist-to-hip-ratio	0.98 ± 0.19	0.95 ± 0.08	1.00 ± 0.16	0.903	0.407	0.373
Fatty liver	0 (0%)	8 (29%)	4 (22%)	**0.001**	**0.004**	0.636
GGT (U/L)	31.9 ± 27.7	48.7 ± 50.2	41.4 ± 64.0	0.093	0.934	0.251
AST (U/L)	21.5 ± 6.4	24.2 ± 17.4	18.9 ± 7.4	0.887	0.070	0.080
ALT (U/L)	21.7 ± 9.3	25.6 ± 24.2	22.2 ± 17.8	0.973	0.321	0.380
FI (µU/mL)	6.7 ± 3.0	7.7 ± 3.8	8.9 ± 6.9	0.461	0.446	0.788
FG (mg/dL)	92.9 ± 6.9	100.3 ± 13.2	109.9 ± 30.0	**0.016**	**0.016**	0.690
HOMA index	1.5 ± 0.7	2.0 ± 1.1	2.6 ± 2.8	0.157	0.069	0.574
Diabetes	0 (0%)	5 (18%)	4 (22%)	**0.009**	**0.004**	0.719
HbA1C (%)	5.4 ± 0.3	6.0 ± 1.7	5.7 ± 0.8	**0.001**	0.103	0.436
CRP (mg/L)	0.17 ± 0.17	0.34 ± 0.35	2.80 ± 6.05	**0.021**	**<0.001**	**0.042**
Ferritin (ng/mL)	112 ± 107	194 ± 284	89 ± 89	0.102	0.290	**0.029**
Hb (g/dL)	14.8 ± 1.1	14.7 ± 1.1	13.1 ± 2.5	0.994	0.155	0.215
TG (mg/L)	103.5 ± 52.7	118.3 ± 59.7	87.1 ± 40.0	0.182	0.569	0.110
HDL–C (mg/L)	60.4 ± 12.3	58.9 ± 12.1	45.7 ± 12.7	0.996	**0.003**	**0.004**
LDL–C (mg/L)	142.5 ± 33.8	141.7 ± 36.7	129.3 ± 37.5	1.000	0.957	0.939
Hypertension	15 (42%)	16 (57%)	7 (39%)	0.223	0.846	0.232
MetS	3 (8%)	3 (11%)	6 (33%)	0.748	**0.021**	0.062

GGT, γ–glutamyl transferase; AST, aspartate aminotransferase; ALT, alanine aminotransferase; FI, fasting insulin; FG, fasting glucose; HOMA index, homeostasis model assessment for insulin resistance; CRP, C-reactive protein; TG, triglyceride; HDL–C/LDL–C, high-density/low-density lipoprotein cholesterol; MetS, metabolic syndrome; data are expressed as means ± standard deviation unless otherwise indicated; *p*-values are assessed by ANOVA (Benjamini–Hochberg or Tamhane post hoc analysis) or Kruskal–Wallis test; *p*-values < 0.05 were considered to indicate statistical significance and are marked in bold.

**Table 2:** Top 30 different metabolites between groups of the training cohort.

Class	Metabolite	Control (*n* = 36)	AA (*n* = 28)	CRC (*n* = 18)	Post Hoc *p*-Value		
					Control vs. AA	Control vs. CRC	AA vs. CRC
PC aa	PC aa C32:2	3.22 ± 0.96	2.60 ± 1.03	1.83 ± 0.76	**0.031**	**<0.001**	**0.028**
	PC aa C32:3	0.29 ± 0.07	0.26 ± 0.07	0.20 ± 0.06	0.229	**<0.001**	**0.012**
	PC aa C34:3	11.08 ± 2.55	10.21 ± 3.29	7.54 ± 2.44	0.099	**<0.001**	**0.006**
	PC aa C34:4	1.40 ± 0.39	1.21 ± 0.52	0.74 ± 0.31	0.226	**<0.001**	**0.002**
	PC aa C36:3	87.51 ± 11.98	86.21 ± 16.29	70.58 ± 16.24	0.990	**0.001**	**0.002**
	PC aa C36:6	0.59 ± 0.18	0.56 ± 0.27	0.31 ± 0.14	0.948	**<0.001**	**<0.001**
	Total PC aa	1039 ± 118	1055 ± 197	870 ± 147	0.969	**0.001**	**0.001**
PC ae	PC ae C38:0	1.40 ± 0.32	1.41 ± 0.50	0.92 ± 0.34	0.665	**<0.001**	**0.001**
	PC ae C40:1	1.22 ± 0.18	1.17 ± 0.34	0.87 ± 0.27	0.159	**<0.001**	**0.003**
PC aa, ae	PUFA PC	940 ± 98	949 ± 167	788 ± 141	0.991	**0.001**	**0.001**
	Total PC	1157 ± 129	1167 ± 210	970 ± 163	0.994	**0.001**	**0.001**
LysoPC	LysoPC a C16:0	128 ± 22	112 ± 22	97 ± 25	**0.032**	**0.027**	0.845
	LysoPC a C17:0	2.35 ± 0.48	1.85 ± 0.56	1.63 ± 0.60	**0.001**	**0.007**	0.980
	LysoPC a C18:0	31 ± 6	26 ± 6	23 ± 8	**0.012**	**0.024**	0.939
	LysoPC a C18:1	25 ± 5	21 ± 5	18 ± 6	**0.034**	**0.033**	0.876
	LysoPC a C18:2	32 ± 9	27 ± 9	21 ± 9	**0.041**	**0.001**	0.167
	LysoPC a C20:3	2.40 ± 0.61	2.06 ± 0.64	1.57 ± 0.5	0.129	**0.019**	0.476
	LysoPC a C28:1	0.49 ± 0.10	0.43 ± 0.13	0.34 ± 0.10	0.233	**0.003**	0.090
	Total lysoPC	233 ± 38	201 ± 38	172 ± 46	**0.007**	**<0.001**	0.056
SM	SM (OH)/SM (non-OH)	0.16 ± 0.02	0.14 ± 0.02	0.14 ± 0.02	**0.020**	**<0.001**	0.358
AC	C2	6.49 ± 1.99	9.16 ± 3.50	8.86 ± 2.65	**<0.001**	**0.001**	0.982
	C12:1	0.04 ± 0.056	0.08 ± 0.06	0.11 ± 0.07	**0.004**	**0.001**	0.215
	C14:1	0.06 ± 0.02	0.09 ± 0.02	0.10 ± 0.04	**0.002**	**0.007**	0.920
	C14:2	0.02 ± 0.01	0.03 ± 0.01	0.03 ± 0.01	**0.033**	0.088	0.990
	C18:1	0.11 ± 0.03	0.13 ± 0.03	0.16 ± 0.04	**0.017**	**<0.001**	0.110
	C2/C0	0.17 ± 0.04	0.24 ± 0.08	0.25 ± 0.08	**<0.001**	**<0.001**	0.514
	(C2 + C3)/C0	0.18 ± 0.04	0.25 ± 0.08	0.26 ± 0.08	**<0.001**	**<0.001**	0.543
	CPT-I-ratio	0.0085 ± 0.002	0.0094 ± 0.002	0.012 ± 0.005	**0.020**	**<0.001**	**0.034**
	Total AC/C0	0.21 ± 0.05	0.30 ± 0.09	0.32 ± 0.09	**<0.001**	**<0.001**	0.322
Amine	Total DMA	0.83 ± 0.12	0.89 ± 0.15	1.01 ± 0.16	0.307	**<0.001**	**0.007**

AC, acylcarnitine; DMA, dimethylarginine; lysoPC, monoacyl-glycerophosphocholine; PC aa, diacyl-glycerophosphocholine; PC ae, alkyl-acyl-glycerophosphocholine; PUFA, poly-unsaturated fatty acid; SM, sphingomyelin; data are expressed in µM (except for ratios) as means ± standard deviation; *p*-values are assessed by ANOVA (Benjamini–Hochberg or Tamhane post hoc analysis) or Kruskal–Wallis test; *p*-values < 0.05 were considered to indicate statistical significance and are marked in bold.

**Table 3:** Baseline characteristics of both validation cohorts.

	Validation CRC (Val. CRC)			Validation AA (Val. AA)			*p*-Value Training Cohort vs.			
	Control (*n* = 29)	CRC (*n* = 48)	*p*-Value	Control (*n* = 28)	AA (*n* = 48)	*p*-Value	Control Val. CRC	Control Val. AA	AA	CRC
Age (years)	68 ± 7	69 ± 10	0.694	66 ± 5	66 ± 10	0.718	**<0.001**	**<0.001**	**0.011**	0.521
Gender (f/m)	26/3	17/31	**<0.001**	0/28	22/26	**<0.001**	**0.001**	**<0.001**	0.727	0.796
BMI (kg/m^2^)	26.3 ± 6.3	26.5 ± 3.7	0.845	28.3 ± 3.7	27.4 ± 3.9	0.357	0.833	**0.005**	0.193	0.416
Waist-to-hip-ratio	0.9 ± 0.01	1.0 ± 0.1	**0.001**	1.00 ± 0.08	1.0 ± 0.1	**0.015**	0.052	**0.033**	0.652	0.760
Fatty liver	10 (37%)	22 (46%)	0.189	17 (61%)	25 (52%)	0.468	**<0.001**	**<0.001**	**0.048**	**0.039**
GGT (U/L)	31.3 ± 29.4	38.3 ± 38.3	0.131	50.1 ± 49.4	46.5 ± 67.5	0.146	0.565	**0.028**	0.354	0.569
AST (U/L)	21.8 ± 6.0	22.7 ± 12.3	0.273	24.8 ± 11.0	22.8 10.4	0.223	0.493	0.233	0.395	0.086
ALT (U/L)	25.9 ± 15.9	22.2 ± 15.9	0.244	26.9 ± 12.8	26.8 ± 21.5	0.280	0.428	0.126	0.456	0.545
FI (µU/mL)	7.9 ± 4.7	8.5 ± 4.7	0.628	12.8 ± 7.1	8.8 ± 4.9	0.103	0.412	**0.004**	0.363	0.427
FG (mg/dL)	105.3 ± 16.2	104.6 ± 18.9	0.797	112.1 ± 23.6	106.1 ± 15.8	0.377	**<0.001**	**<0.001**	0.034	0.749
HOMA index	2.5 ± 1.6	2.3 ± 1.5	0.505	3.8 ± 2.5	2.4 ± 1.5	0.155	**0.022**	**0.003**	0.275	0.650
Diabetes	4 (14%)	9 (19%)	0.620	7 (25%)	4 (8%)	**0.048**	**0.020**	**0.002**	0.218	0.754
HbA1C (%)	5.7 ± 0.4	6.0 ± 0.9	0.307	5.9 ± 0.6	5.9 ± 0.6	0.824	**<0.001**	**<0.001**	0.321	0.180
CRP (mg/L)	0.64 ± 2.39	1.14 ± 1.86	**<0.001**	0.52 ± 0.92	0.46 ± 0.87	0.290	0.311	**<0.001**	0.741	0.432
Ferritin (ng/mL)	161 ± 115	110 ± 96	**0.043**	297 ± 236	224 ± 230	0.063	**0.045**	**<0.001**	0.162	0.492
Hb (g/dL)	14.5 ± 1.2	13.9 ± 2.2	0.553	15.2 ± 1.4	16.7 ± 14.5	**0.024**	0.319	0.057	0.970	0.152
TG (mg/L)	107.9 ± 44.2	121.6 ± 57.9	0.386	136.7 ± 58.7	125.7 ± 69.6	0.274	0.253	**0.015**	0.575	**0.041**
HDL-C (mg/L)	66.3 ± 18.6	57.5 ± 11.7	**0.030**	52.5 ± 12.7	64.0 ± 16.1	**0.004**	0.313	**0.020**	0.209	**0.002**
LDL-C (mg/L)	139.7 ± 36.1	134.4 ± 39.0	0.561	147.8 ± 43.3	145.7 ± 38.0	0.828	0.957	0.713	0.876	0.624
Hypertension	14 (50%)	31 (65%)	0.215	14 (50%)	23 (48%)	0.862	0.510	0.510	0.441	0.062
MetS	10 (36%)	15 (31%)	0.691	13 (46%)	14 (29%)	0.132	**0.007**	**0.001**	0.064	0.872

GGT, γ–glutamyl transferase; AST, aspartate aminotransferase; ALT, alanine aminotransferase; FI, fasting insulin; FG, fasting glucose; HOMA index, homeostasis model assessment for insulin resistance; CRP, C-reactive protein; TG, triglyceride; HDL–C/LDL–C, high-density/low-density lipoprotein cholesterol; MetS, metabolic syndrome; data are expressed as means ± standard deviation unless otherwise indicated; *p*-values are assessed by ANOVA (Benjamini–Hochberg or Tamhane post hoc analysis) or Kruskal–Wallis test; *p*-values < 0.05 were considered to indicate statistical significance and are marked in bold.

**Table 4:** Top 10 metabolites of the AA and CRC validation cohorts.

		**Validation CRC**		
**Class**	**Metabolite**	**Control** **(*n* = 29)**	**CRC** **(*n* = 48)**	***p*-Value**
PC aa	PC aa C32:3	0.44 ± 0.14	0.33 ± 0.09	**<0.001**
PC ae	PC ae C36:2	14.60 ± 3.44	11.52 ± 3.51	**<0.001**
LysoPC	LysoPC a C17:0	3.01 ± 0.76	2.31 ± 0.77	**<0.001**
	LysoPC a C24:0	0.22 ± 0.10	0.17 ± 0.10	**<0.001**
	LysoPC a C28:0	0.80 ± 0.27	0.24 ± 0.33	**0.002**
	LysoPC a C28:1	0.65 ± 0.27	0.48 ± 0.24	**0.001**
SM	SM (OH) C22:1	19.27 ± 4.18	16.12 ± 4.60	**0.001**
	SM (OH) C22:2	19.10 ± 3.48	15.50 ± 4.85	**0.001**
	SM (OH)/SM (non-OH)	0.17 ± 0.02	0.15 ± 0.03	**<0.001**
	Total SM (OH)	54.86 ± 10.38	45.62 ± 12.96	**<0.001**
		**Validation AA**		
**Class**	**Metabolite**	**Control** **(*n* = 28)**	**AA** **(*n* = 48)**	***p*-Value**
PC aa	PC aa C38:6	60.44 ± 18.06	76.49 ± 20.76	**0.001**
PC ae	PC ae C38:6	5.92 ± 1.53	7.19 ± 1.60	**0.001**
	PC ae C40:6	4.23 ± 1.02	5.05 ± 1.00	**0.001**
SM	SM C18:1	12.19 ± 3.37	14.72 ± 3.04	**0.001**
	SM C24:1	55.63 ± 12.91	64.34 ± 10.72	**0.002**
	SM (OH) C22:2	13.51 ± 3.54	16.33 ± 3.51	**0.001**
	SM (OH) C24:1	1.45 ± 0.35	1.71 ± 0.37	**0.003**
	Total SM	308.06 ± 61.13	347.89 ± 49.01	**0.003**
	Total SM (OH)	40.08 ± 10.05	47.17 ± 9.52	**0.003**
AA	Cit/Arg	0.22 ± 0.06	0.27 ± 0.07	**0.001**

AA, amino acid; LysoPC, monoacyl-glycerophosphocholine; PC aa, diacyl-glycerophosphocholine; PC ae, alky-acyl-glycerophosphocholine; SM, sphingomyelin; data are expressed in µM (except for ratios) as means ± standard deviation; *p*-values are assessed by *t*-test or Mann–Whitney U test; *p*-values < 0.05 were considered to indicate statistical significance and are marked in bold.

## Data Availability

Data is contained within the article and the supplementary material.

## Supplementary Materials

The following supporting information can be downloaded at: https://www.mdpi.com/article/10.3390/jcm11030721/s1, Table S1: List of metabolites of the metabolomics analysis of the training cohort, Table S2: Top 10 metabolites positive and negative correlated with control to adenoma to carcinoma sequence, Table S3: Top 10 metabolites positive and negative correlated with the total amount of DMA, Table S4: Top 10 metabolites positive and negative correlated with kynurenine to tryptophan ratio, Table S5: Summary of top 10 metabolites derived from single ROC analysis, Table S6: List of metabolites of the metabolomics analysis of the both validation cohorts, Figure S1: Sparse partial least discriminant analysis (sPLS-DA) of the two validation cohorts, Figure S2: Selected validated metabolites of the training cohort and the validation cohorts.
