# Supplementary material for: Changing Metabolic Patterns along the Colorectal Adenoma–Carcinoma Sequence
Source: J Clin Med. 2022 Jan 29;11(3):721. doi: 10.3390/jcm11030721 (PMC8836789; doi:10.3390/jcm11030721)
Supplement: Supplementary file 1 [file jcm-11-00721-s001.zip › jcm-1547146-supplementary.pdf]

Journal of Clinical Medicine

Supplementary Materials: Changing metabolic Patterns along the Colorectal Adenoma-Carcinoma Sequence

Julia Tevini, Sebastian K. Eder, Ursula Huber-Schönauer, David Niederseer, Georg Strebinger, Johanna M. Gostner, Elmar Aigner, Christian Datz and Thomas K. Felder

Table S1. List of metabolites of the metabolomics analysis of the training cohort.

	Training cohort (n=82)					
				Post hoc P-value		
(µM)	Ctrl (n=36)	AA (n=28)	CRC (n=18)	Ctrl vs. AA	Ctrl vs. CRC	AA vs. CRC
**Acylcarnitines**						
C0	39 ± 7	38 ± 7	36 ± 8	0.875	0.970	0.999
C2	6.49 ± 1.99	9.16 ± 3.50	8.86 ± 2.65	**<0.001**	**0.001**	0.982
C3	0.34 ± 0.11	0.35 ± 0.09	0.33 ± 0.19	0.350	0.267	0.062
C4	0.19 ± 0.08	0.21 ± 0.09	0.18 ± 0.06	0.256	0.646	0.322
C5	0.12 ± 0.06	0.13 ± 0.05	0.12 ± 0.07	0.797	0.985	0.770
C6 (C4:1-DC)	0.02 ± 0.03	0.05 ± 0.05	0.05 ± 0.05	**0.003**	**0.004**	0.749
C7-DC	0.022 ± 0.022	0.031 ± 0.022	0.038 ± 0.020	0.158	**0.025**	0.262
C8	0.13 ± 0.11	0.20 ± 0.12	0.21 ± 0.07	**0.006**	**0.004**	0.628
C9	0.04 ± 0.02	0.03 ± 0.03	0.03 ± 0.02	0.340	0.549	0.734
C10	0.23 ± 0.11	0.30 ± 0.12	0.32 ± 0.11	**0.004**	**0.003**	0.521
C10:1	0.07 ± 0.08	0.14 ± 0.07	0.13 ± 0.07	**0.002**	**0.008**	0.839
C10:2	0.01 ± 0.02	0.01 ± 0.02	0.01 ± 0.02	0.136	0.892	0.218
C12	0.05 ± 0.07	0.10 ± 0.07	0.13 ± 0.08	**0.019**	**0.002**	0.184
C12:1	0.04 ± 0.05	0.08 ± 0.06	0.11 ± 0.07	**0.004**	**0.001**	0.215
C14	0.03 ± 0.01	0.04 ± 0.01	0.04 ± 0.01	**0.031**	**0.032**	0.693
C14:1	0.06 ± 0.02	0.09 ± 0.02	0.10 ± 0.04	**0.002**	**0.007**	0.920
C14:1-OH	0.003 ± 0.007	0.0051 ± 0.0082	0.0056 ± 0.0095	0.269	0.262	0.821
C14:2	0.02 ± 0.01	0.03 ± 0.01	0.03 ± 0.01	**0.033**	0.088	0.990
C16	0.11 ± 0.02	0.11 ± 0.03	0.12 ± 0.03	0.482	0.137	0.651
C16:1	<LOQ	0.003 ± 0.014	0.0131 ± 0.0304	0.257	**0.013**	0.117
C16:1-OH	0.001 ± 0.004	0.0035 ± 0.0061	0.0026 ± 0.0059	0.074	0.305	0.651
C16:2	0.0003 ± 0.002	0.0025 ± 0.0054	0.0053 ± 0.0080	**0.036**	**0.001**	0.190
C18	0.06 ± 0.01	0.056 ± 0.01	0.06 ± 0.02	0.776	0.295	0.260
C18:1	0.11 ± 0.03	0.13 ± 0.03	0.16 ± 0.04	**0.017**	**<0.001**	0.110
C18:2	0.05 ± 0.01	0.05 ± 0.01	0.06 ± 0.02	0.171	0.054	0.521
**Amino acids**						
Ala	381 ± 68	379 ± 65	367 ± 55	0.901	0.487	0.790
Arg	112 ± 24	111 ± 21	126 ± 25	0.967	0.056	0.114
Asn	53 ± 7	51 ± 8	51 ± 10	0.403	0.288	0.920
Asp	42 ± 10	42 ± 10	46 ± 18	0.825	0.875	0.690
Cit	37 ± 8	37 ± 8	35 ± 12	0.951	0.790	0.556
Gln	640 ± 93	606 ± 73	608 ± 156	0.180	0.125	0.451
Glu	71 ± 19	85 ± 29	83 ± 35	0.177	0.541	1.000

Gly	335 ± 86	293 ± 64	295 ± 64	**0.036**	0.132	0.710
His	85 ± 9	81 ± 11	77 ± 21	0.288	**0.004**	0.083
Ile	74 ± 13	84 ± 25	84 ± 25	0.120	0.158	0.946
Leu	164 ± 27	175 ± 39	169 ± 48	0.440	0.912	0.536
Lys	244 ± 39	244 ± 40	232 ± 54	0.978	0.166	0.137
Met	24 ± 4	23 ± 4	25 ± 5	0.735	0.441	0.356
Orn	86 ± 22	94 ± 17	81 ± 24	**0.045**	0.987	0.320
Phe	69 ± 10	72 ± 9	74 ± 18	0.120	0.971	0.411
Pro	178 ± 54	167 ± 42	160 ± 49	0.436	0.248	0.581
Ser	156 ± 36	143 ± 22	159 ± 39	0.234	0.854	0.173
Thr	123 ± 26	127 ± 22	111 ± 36	0.503	0.079	0.050
Trp	67 ± 11	67 ± 14	62 ± 16	0.994	1.000	0.996
Tyr	65 ± 13	69 ± 11	68 ± 19	0.412	0.743	0.983
Val	225 ± 36	232 ± 44	223 ± 47	0.715	0.388	0.405
**Biogenic amines**						
AcOrn	1.38 ± 0.72	1.09 ± 0.56	1.03 ± 0.66	0.120	0.088	0.581
ADMA	0.53 ± 0.11	0.58 ± 0.10	0.58 ± 0.11	0.348	0.159	0.801
Alpha AAA	1.15 ± 0.30	1.37 ± 0.63	1.20 ± 0.36	0.398	0.551	0.702
Creatinine	83 ± 17	86 ± 22	95 ± 27	0.617	0.108	0.290
Histamine	0.35 ± 0.003	0.35 ± 0.006	0.35 ± 0.003	0.322	0.080	0.406
Kynurenine	2.78 ± 0.58	2.95 ± 0.89	3.62 ± 2.21	0.607	0.393	0.677
MetSO	0.61 ± 0.23	0.69 ± 0.21	0.78 ± 0.51	0.064	0.202	0.884
Putrescine	0.12 ± 0.05	0.13 ± 0.08	0.11 ± 0.05	0.860	0.811	0.628
Serotonin	0.63 ± 0.31	0.65 ± 0.36	0.72 ± 0.40	0.730	0.287	0.574
Spermidine	0.23 ± 0.05	0.24 ± 0.07	0.23 ± 0.04	0.771	0.941	0.796
Spermine	0.21 ± 0.02	0.21 ± 0.02	0.21 ± 0.02	0.326	0.693	0.839
Taurine	132 ± 43	123 ± 33	110 ± 37	0.461	0.095	0.246
Total DMA	0.83 ± 0.12	0.89 ± 0.15	1.01 ± 0.16	0.307	**<0.001**	**0.007**
t4-OH-Pro	11.26 ± 5.83	11.84 ± 6.06	9.58 ± 3.59	0.590	0.505	0.246
**Glycerophospholipids**						
LysoPC a C16:0	128 ± 22	112 ± 22	97 ± 25	**0.032**	**0.027**	0.845
LysoPC a C16:1	3.57 ± 0.80	3.30 ± 1.01	2.46 ± 1.10	0.463	**0.027**	0.253
LysoPC a C17:0	2.35 ± 0.48	1.85 ± 0.56	1.63 ± 0.60	**0.001**	**0.007**	0.980
LysoPC a C18:0	31 ± 6	26 ± 6	23 ± 8	**0.012**	**0.024**	0.939
LysoPC a C18:1	25 ± 5	21 ± 5	18 ± 6	**0.034**	**0.033**	0.876
LysoPC a C18:2	32 ± 9	27 ± 9	21 ± 9	**0.041**	**0.001**	0.167
LysoPC a C20:3	2.40 ± 0.61	2.06 ± 0.64	1.57 ± 0.56	0.129	**0.019**	0.476
LysoPC a C20:4	7.85 ± 2.08	6.92 ± 2.04	6.08 ± 2.38	0.187	0.790	0.925
LysoPC a C24:0	0.19 ± 0.04	0.19 ± 0.04	0.17 ± 0.04	0.974	0.181	0.305
LysoPC a C26:0	0.27 ± 0.09	0.25 ± 0.09	0.22 ± 0.06	0.444	**0.039**	0.150
LysoPC a C26:1	0.13 ± 0.03	0.13 ± 0.03	0.11 ± 0.02	0.999	0.634	0.565
LysoPC a C28:0	0.37 ± 0.07	0.32 ± 0.10	0.27 ± 0.12	**0.019**	**0.001**	0.173
LysoPC a C28:1	0.49 ± 0.10	0.43 ± 0.13	0.34 ± 0.10	0.233	**0.003**	0.090
PC aa C24:0	0.07 ± 0.03	0.07 ± 0.03	0.06 ± 0.02	0.631	0.142	0.478
PC aa C28:1	2.63 ± 0.55	2.47 ± 0.62	2.09 ± 0.55	0.659	**0.006**	0.088
PC aa C30:0	3.66 ± 0.91	3.28 ± 1.34	2.54 ± 0.76	0.076	**<0.001**	**0.046**

PC aa C32:0	8.44 ± 1.45	8.68 ± 2.14	7.69 ± 1.53	0.905	0.396	0.177
PC aa C32:1	10.15 ± 4.04	10.48 ± 5.27	6.72 ± 2.03	0.925	**0.001**	**0.007**
PC aa C32:2	3.22 ± 0.96	2.60 ± 1.03	1.83 ± 0.76	**0.031**	**<0.001**	**0.028**
PC aa C32:3	0.29 ± 0.07	0.26 ± 0.07	0.20 ± 0.06	0.229	**<0.001**	**0.012**
PC aa C34:1	139 ± 33	141 ± 35	119 ± 20	0.529	**0.033**	**0.011**
PC aa C34:2	230 ± 32	229 ± 37	198 ± 37	0.551	**0.003**	**0.024**
PC aa C34:3	11.08 ± 2.55	10.21 ± 3.29	7.54 ± 2.44	0.099	**<0.001**	**0.006**
PC aa C34:4	1.40 ± 0.39	1.21 ± 0.52	0.74 ± 0.31	0.226	**<0.001**	**0.002**
PC aa C36:0	1.33 ± 0.32	1.29 ± 0.45	1.00 ± 0.32	0.375	**0.003**	**0.042**
PC aa C36:1	29.81 ± 6.14	28.68± 6.74	23.86 ± 5.29	0.891	**0.005**	**0.036**
PC aa C36:2	139 ± 21	134 ± 25	117 ± 26	0.796	**0.004**	**0.045**
PC aa C36:3	87.51 ± 11.98	86.21 ± 16.29	70.58 ± 16.24	0.990	**0.001**	**0.002**
PC aa C36:4	122 ± 22	128 ± 35	104 ± 33	0.842	0.133	0.075
PC aa C36:5	17.44 ± 5.98	19.95 ± 13.27	10.14 ± 3.82	0.660	**<0.001**	**<0.001**
PC aa C36:6	0.59 ± 0.18	0.56 ± 0.27	0.31 ± 0.14	0.948	**<0.001**	**<0.001**
PC aa C38:0	2.20 ± 0.46	2.16 ± 0.64	1.84 ± 0.45	0.457	**0.036**	0.224
PC aa C38:1	1.09 ± 0.20	1.12 ± 0.31	0.92 ± 0.22	0.925	**0.011**	**0.011**
PC aa C38:3	31 ± 6	32 ± 9	25 ± 7	0.849	**0.043**	**0.011**
PC aa C38:4	77 ± 16	83 ± 26	69 ± 21	0.570	**0.043**	0.056
PC aa C38:5	36 ± 5	38 ± 13	28 ± 8	0.538	**<0.001**	**0.002**
PC aa C38:6	53 ± 12	58 ± 20	44 ± 12	0.429	**0.030**	**0.016**
PC aa C40:1	0.07 ± 0.14	0.06 ± 0.14	0.04 ± 0.11	0.512	0.357	0.701
PC aa C40:2	0.23 ± 0.03	0.24 ± 0.07	0.20 ± 0.05	0.776	**0.033**	0.120
PC aa C40:3	0.35 ± 0.05	0.37 ± 0.12	0.30 ± 0.06	0.685	**0.009**	**0.016**
PC aa C40:4	2.94 ± 0.50	2.95 ± 0.80	2.47 ± 0.53	0.998	**0.016**	0.055
PC aa C40:5	6.93 ± 1.28	7.33 ± 2.21	5.69 ± 1.10	0.760	**0.003**	**0.005**
PC aa C40:6	18 ± 5	20 ± 8	16 ± 4	0.203	0.171	**0.037**
PC aa C42:0	0.44 ± 0.09	0.43 ± 0.08	0.39 ± 0.11	0.965	0.112	0.270
PC aa C42:1	0.25 ± 0.05	0.24 ± 0.05	0.22 ± 0.06	0.803	**0.030**	0.190
PC aa C42:2	0.19 ± 0.02	0.19 ± 0.04	0.16 ± 0.04	1.000	**0.004**	**0.008**
PC aa C42:4	0.14 ± 0.02	0.14 ± 0.02	0.113 ± 0.03	0.952	0.064	0.190
PC aa C42:5	0.29 ± 0.05	0.33 ± 0.15	0.26 ± 0.06	0.144	0.069	**0.013**
PC aa C42:6	0.37 ± 0.06	0.37 ± 0.12	0.27 ± 0.07	0.547	**<0.001**	**0.001**
PC ae C30:0	0.33 ± 0.07	0.27 ± 0.09	0.24 ± 0.08	**0.024**	**0.002**	0.576
PC ae C30:1	0.19 ± 0.07	0.17 ± 0.06	0.14 ± 0.05	0.746	**0.038**	0.260
PC ae C30:2	0.08 ± 0.02	0.08 ± 0.02	0.07 ± 0.02	0.724	0.106	0.511
PC ae C32:1	1.85 ± 0.40	1.84 ± 0.44	1.64 ± 0.50	1.000	0.291	0.345
PC ae C32:2	0.43 ± 0.10	0.43 ± 0.09	0.37 ± 0.11	1.000	0.146	0.146
PC ae C34:0	1.06 ± 0.21	0.99 ± 0.25	0.87 ± 0.24	0.616	**0.026**	0.272
PC ae C34:1	5.93 ± 1.1	5.66 ± 1.11	5.13 ± 1.03	0.762	0.056	0.323
PC ae C34:2	8.52 ± 1.73	7.44 ± 1.89	6.96 ± 2.21	0.071	**0.016**	0.793
PC ae C34:3	5.60 ± 1.40	4.99 ± 1.75	4.26 ± 1.63	0.034	**0.011**	0.333
PC ae C36:0	0.64 ± 0.11	0.64 ± 0.13	0.59 ± 0.11	0.997	0.272	0.393
PC ae C36:1	4.61 ± 0.90	4.27 ± 0.89	3.68 ± 0.92	0.419	**0.002**	0.095
PC ae C36:2	9.40 ± 1.93	8.53 ± 1.69	7.7 ± 2.10	0.226	**0.010**	0.399
PC ae C36:3	5.03± 0.95	4.54 ± 1.12	4.20 ± 1.24	0.200	**0.026**	0.647

PC ae C36:4	13.4 ± 3.0	12.5 ± 3.1	11.3 ± 2.3	0.462	**0.036**	0.449
PC ae C36:5	8.29 ± 2.18	8.16 ± 2.40	6.52 ± 1.95	0.993	**0.022**	0.049
PC ae C38:0	1.40 ± 0.32	1.41 ± 0.50	0.92 ± 0.34	0.665	**<0.001**	**0.001**
PC ae C38:1	0.79 ± 0.14	0.72 ± 0.15	0.64 ± 0.20	0.311	**0.008**	0.282
PC ae C38:2	1.43 ± 0.26	1.32 ± 0.25	1.19 ± 0.34	0.312	**0.012**	0.338
PC ae C38:3	3.09 ± 0.56	2.93 ± 0.56	2.63 ± 0.72	0.745	**0.042**	0.276
PC ae C38:4	9.40 ± 1.88	8.98 ± 1.67	8.66 ± 1.77	0.762	0.433	0.915
PC ae C38:5	13.4 ± 2.4	13.1 ± 2.7	12.1 ± 2.5	0.892	0.173	0.491
PC ae C38:6	5.08 ± 1.02	5.04 ± 1.38	4.02 ± 1.02	0.612	**0.003**	**0.011**
PC ae C40:1	1.22 ± 0.18	1.17 ± 0.34	0.87 ± 0.27	0.159	**<0.001**	**0.003**
PC ae C40:2	1.34 ± 0.28	1.36 ± 0.27	1.18 ± 0.31	0.991	0.156	0.115
PC ae C40:3	0.84 ± 0.16	0.80 ± 0.13	0.78 ± 0.23	0.631	0.112	0.115
PC ae C40:4	1.73 ± 0.35	1.68 ± 0.27	1.67 ± 0.41	0.926	0.898	0.999
PC ae C40:5	2.84 ± 0.50	2.84 ± 0.47	2.65 ± 0.58	1.000	0.521	0.565
PC ae C40:6	3.20 ± 0.56	3.14 ± 0.70	2.75 ± 0.57	0.961	**0.036**	0.112
PC ae C42:1	0.30 ± 0.03	0.29 ± 0.06	0.26 ± 0.06	0.684	**0.036**	0.242
PC ae C42:2	0.56 ± 0.08	0.51 ± 0.11	0.45 ± 0.11	0.279	**0.001**	0.083
PC ae C42:3	0.64 ± 0.11	0.61 ± 0.13	0.52 ± 0.13	0.866	**0.006**	0.047
PC ae C42:4	0.82 ± 0.18	0.81 ± 0.14	0.79 ± 0.23	0.999	0.956	0.972
PC ae C42:5	1.81 ± 0.34	1.83 ± 0.30	1.75 ± 0.35	0.987	0.922	0.814
PC ae C44:3	0.09 ± 0.02	0.09 ± 0.02	0.08 ± 0.02	0.839	**0.019**	0.122
PC ae C44:4	0.39 ± 0.08	0.38 ± 0.07	0.35 ± 0.09	1.000	0.235	0.300
PC ae C44:5	1.61 ± 0.39	1.62 ± 0.36	1.49 ± 0.37	0.995	0.643	0.550
PC ae C44:6	1.09 ± 0.25	1.07 ± 0.20	1.00 ± 0.34	0.970	0.707	0.844
**Sphingomyelins**						
SM (OH) C14:1	5.89 ± 1.30	5.4 ± 1.32	5.04 ± 1.13	0.419	0.071	0.662
SM (OH) C16:1	2.49 ± 0.55	2.49 ± 0.56	2.36 ± 0.59	1.000	0.828	0.839
SM (OH) C22:1	9.79 ± 1.97	9.17 ± 1.79	7.74 ± 2.00	0.109	**0.002**	**0.022**
SM (OH) C22:2	7.87 ± 1.53	7.29 ± 1.64	6.21 ± 1.63	0.444	**0.002**	0.082
SM (OH) C24:1	1.01 ± 0.27	0.95 ± 0.23	0.79 ± 0.21	0.297	**0.007**	0.060
SM C16:0	79 ± 13	78 ± 9	76 ± 10	0.969	0.633	0.807
SM C16:1	12.9 ± 2.3	13.0 ± 2.4	11.7 ± 2.4	0.985	0.269	0.185
SM C18:0	15 ± 3	17 ± 4	16 ± 3	0.068	0.731	0.647
SM C18:1	7.02± 1.60	7.95 ± 1.98	7.13 ± 2.01	**0.032**	0.927	0.105
SM C20:2	0.31 ± 0.07	0.33 ± 0.09	0.30 ± 0.08	0.551	0.633	0.275
SM C22:3	0.30 ± 0.14	0.41 ± 0.14	0.35 ± 0.16	**0.010**	0.494	0.470
SM C24:0	14.6 ± 2.9	14.6 ± 2.6	11.7 ± 2.6	0.968	**0.001**	**0.001**
SM C24:1	40.4 ± 6.84	42.9 ± 8.53	38.3 ± 5.43	0.416	0.707	0.112
SM C26:0	0.17 ± 0.05	0.16 ± 0.04	0.13 ± 0.04	0.354	**0.008**	0.059
SM C26:1	0.35 ± 0.08	0.38 ± 0.09	0.31 ± 0.07	0.423	0.208	**0.015**
**Monosaccharides**						
H1	4003 ± 346	4363 ± 674	4734 ± 1321	**0.039**	**0.015**	0.458
**Ratios and sums**						
**Acylcarnitines**						
C2/C0	0.170 ± 0.045	0.239 ± 0.079	0.254 ± 0.076	**<0.001**	**<0.001**	0.514
(C2+C3)/C0	0.178 ± 0.045	0.248 ± 0.079	0.263 ± 0.077	**<0.001**	**<0.001**	0.543

CPT-I-ratio	0.009 ± 0.002	0.009 ± 0.002	0.012 ± 0.005	**0.020**	**<0.001**	**0.034**
Total AC/C0	0.214 ± 0.053	0.295 ± 0.089	0.320 ± 0.092	**<0.001**	**<0.001**	0.322
Total AC-DC/Total AC	0.0011 ± 0.0013	0.0017 ± 0.0015	0.0025 ± 0.0015	0.089	**0.001**	0.068
Total AC-OH/Total AC	0.004 ± 0.0012	0.0003 ± 0.0006	0.0008 ± 0.0016	0.786	0.656	0.766
**Amino acids and biogenic amines**						
ADMA/Arg	0.005 ± 0.002	0.005 ± 0.002	0.005 ± 0.001	0.213	0.869	0.156
Aromatic AA	201 ± 29	208 ± 28	204 ± 47	0.317	0.920	0.405
BCAA	463 ± 72	491 ± 102	476 ± 114	0.417	0.797	0.444
Calc. SDMA/Arg	0.003 ± 0.001	0.003 ± 0.001	0.004 ± 0.002	0.992	0.776	0.653
Cit/Arg	0.341 ± 0.084	0.345 ± 0.088	0.290 ± 0.136	1.000	**0.048**	**0.046**
Cit/Orn	0.453 ± 0.122	0.406 ± 0.103	0.443 ± 0.126	0.172	0.820	0.891
Essential AA	1076 ± 120	1106 ± 161	1056 ± 219	0.507	0.226	0.192
Fischer ratio	2.326 ± 0.345	2.352 ± 0.335	2.358 ± 0.410	0.871	0.941	0.928
Glucogenic AA	872 ± 139	815 ± 80	820 ± 132	0.121	0.668	0.936
Kyn/Trp	0.042 ± 0.008	0.044 ± 0.012	0.060 ± 0.033	0.561	**0.038**	0.132
MetSO/Met	0.025 ± 0.009	0.030 ± 0.009	0.031 ± 0.017	**0.027**	0.215	0.761
Non-essential AA	2034 ± 242	1947 ± 165	1961 ± 339	0.076	0.158	0.685
Orn/Arg	0.80 ± 0.26	0.88 ± 0.25	0.68 ± 0.29	0.137	**0.045**	0.002
Putrescine/Orn	0.0015 ± 0.0008	0.0014 ± 0.0008	0.0016 ± 0.0010	0.766	0.783	0.644
Ser/Trp	0.0036 ± 0.0010	0.0038 ± 0.0015	0.0040 ± 0.0014	0.946	0.322	0.431
Spermidine/Putrescine	2.28 ± 1.11	2.19 ± 0.94	2.25 ± 1.23	0.803	0.739	0.895
Spermine/Spermidine	0.92 ± 0.14	0.93 ± 0.23	0.92 ± 0.12	0.892	0.811	0.804
Total AA	3232 ± 326	3183 ± 282	3134 ± 572	0.499	0.158	0.301
Total DMA/Arg	0.008 ± 0.002	0.008 ± 0.002	0.008 ± 0.003	0.218	0.369	0.839
Tyr/Phe	0.936 ± 0.136	0.966 ± 0.138	0.918 ± 0.123	0.868	1.000	0.941
**Lipids**						
MUFA PC	198 ± 43	199 ± 46	166 ± 28	0.745	**0.007**	**0.004**
MUFA PC/SFA PC	10.16 ± 1.71	10.37 ± 1.20	10.42 ± 1.50	0.941	0.920	0.999
PUFA PC	940 ± 98	949 ± 167	788 ± 141	0.991	**0.001**	**0.001**
PUFA PC/MUFA PC	4.89 ± 0.82	4.88 ± 0.63	4.80 ± 0.76	0.850	0.474	0.368
PUFA PC/SFA PC	48.6 ± 5.1	50.3 ± 6.8	49.5 ± 7.2	0.695	0.961	0.969
SFA PC	19.6 ± 3.2	19.3 ± 4.6	16.1 ± 3.1	0.988	**0.007**	**0.021**
Total lysoPC	233 ± 38	201 ± 38	172 ± 46	**0.007**	**<0.001**	0.056
Total lysoPC/total PC	5.07 ± 0.91	5.94 ± 1.32	5.90 ± 1.38	**0.005**	**0.029**	0.605
Total PC	1157 ± 129	1167 ± 210	970 ± 163	0.994	**0.001**	**0.001**
Total PC aa	1039 ± 118	1055 ± 197	870 ± 147	0.969	**0.001**	**0.001**
Total PC ae	119 ± 18	112 ± 18	100 ± 21	0.462	**0.004**	0.113
Total (PC+SM)	1355 ± 153	1368 ± 230	1154 ± 184	0.991	**0.001**	**0.001**
Total SM	198 ± 33	201 ± 29	184 ± 27	0.969	0.335	0.203
Total SM (non-OH)	171 ± 28	176 ± 25	162 ± 23	0.854	0.586	0.253
Total SM (OH)	27.04 ± 5.24	25.33 ± 5.06	22.14 ± 5.21	0.506	**0.006**	0.129
Total SM (OH)/ total SM (non-OH)	0.158 ± 0.016	0.144 ± 0.021	0.136 ± 0.021	**0.020**	**<0.001**	0.358
Total SM/total PC	0.171 ± 0.021	0.175 ± 0.022	0.192 ± 0.021	0.879	**0.004**	**0.027**
Total SM/total PL pool	0.124 ± 0.014	0.129 ± 0.013	0.140 ± 0.013	0.441	**<0.001**	**0.028**

AA, amino acid; AC, acylcarnitine; AC-DC, dicarboxyacylcarnitine; AC-OH, hydroxylated acylcarnitine; Arg, arginine; BCAA, branched chain amino acid; Cit, citrulline; DMA, dimethylarginine; Kyn, kynurenine; lysoPC, monoacyl-glycerophosphocholine;

Met, methionine; MetSO, methionine sulfoxide; MUFA; mono-unsaturated fatty acid; Orn, ornithine; PC aa, diacyl-glycerophosphocholine; PC ae, alkyl-acyl-glycerophosphocholine; Phe, phenylalanine; PL, phospholipid; PUFA, polyunsaturated fatty acid; SFA, saturated fatty acid; Ser, serine; SM, sphingomyelin; Trp, tryptophan; Tyr, tyrosine; data are expressed in µM as means ± standard deviation; P-values are assessed by ANOVA (Benjamini-Hochberg or Tamhane post hoc analysis) or Kruskal-Wallis test; P-values <0.05 were considered to indicate statistical significance and are marked in bold; metabolites are sorted in alphabetical order.

Table S2. Top 10 metabolites positive and negative correlated with control to adenoma to carcinoma sequence.

**Positive correlation**	Spearman's rank correlation factor		**Negative correlation**
Total AC/C0	0.544	-0.516	PC aa C34:4
C2/C0	0.526	-0.503	PC aa C32:2
(C2+C3)/C0	0.526	-0.498	Total lysoPC
C14:1	0.499	-0.475	LysoPC a C17:0
C18:1	0.455	-0.474	LysoPC a C18:2
C14:2	0.431	-0.459	PC aa C36:6
CPT-I-ratio	0.430	-0.459	PC aa C32:3
C2	0.430	-0.458	LysoPC a C18:0
Total DMA	0.427	-0.450	PC ae C40:1
C12:1	0.409	-0.447	PC aa C34:3

AC, acylcarnitine; DMA, dimethylarginine; lysoPC, monoacyl-glycerophosphocholine; PC aa, diacyl-glycerophosphocholine.

Table S3. Top 10 metabolites positive and negative correlated with the total amount of DMA.

**Positive correlation**	Spearman's rank correlation factor		**Negative correlation**
Calc. SDMA	0.653	-0.468	LysoPC a C18:2
Total DMA/Arg	0.595	-0.417	Total lysoPC
Calc. SDMA/Arg	0.593	-0.398	LysoPC a C18:0
C18:1	0.480	-0.380	LysoPC a C20:3
ADMA	0.461	-0.360	PC aa C34:4
CPT-I-ratio	0.456	-0.356	PC aa C32:2
Total AC/C0	0.446	-0.336	PC aa C32:3
C6 (C4:1-DC)	0.439	-0.334	LysoPC a C28:0
Total AC-DC/Total AC	0.424	-0.329	PC aa C36:6
C2/C0	0.418	-0.322	LysoPC a C16:0

AC, acylcarnitine; DMA, dimethylarginine; lysoPC, monoacyl-glycerophosphocholine; PC aa, diacyl-glycerophosphocholine; SDMA, symmetric dimethylarginine.

Table S4. Top 10 metabolites positive and negative correlated with kynurenine to tryptophan ratio.

**Positive correlation**	Spearman's rank correlation factor		**Negative correlation**
Kynurenine	0.717	-0.438	PC aa C24:0
Creatinine	0.395	-0.434	LysoPC a C26:1
Total SM/Total PL pool	0.377	-0.422	LysoPC a C24:0
Calc. SDMA	0.373	-0.379	LysoPC a C26:0
Calc. SDMA/Arg	0.353	-0.374	LysoPC a C16:0
Total AC/C0	0.343	-0.339	PC aa C34:4
Total SM/Total PC	0.333	-0.329	LysoPC a C16:1
C2	0.325	-0.320	Total lysoPC

CPT-I-ratio	0.324	-0.307	PC ae C44:3
C2/C0	0.323	-0.300	PC aa C36:4

AC, acylcarnitine; lysoPC, monoacyl-glycerophosphocholine; PC aa, diacyl-glycerophosphocholine; SDMA, symmetric dimethylarginine; SM, sphingomyelin.

Table S5. Summary of top 10 metabolites derived from single ROC analysis, based on area under the curve values (AUC) as percentages.

Ctrl vs. CRC	AUC (%)	C.I. (95%)	Ctrl vs. AA	AUC (%)	C.I. (95%)
PC aa C34:4	90.7	82.4-97.0	(C2+C3)/C0	78.7	65.4-89.0
PC aa C36:6	89.7	80.6-97.0	Total AC/C0	78.4	65.7-89.1
PC aa C32:2	87.3	77.2-95.4	C2/C0	78.3	66.2-88.3
Total AC/C0	86.7	75.3-95.1	C14:1	76.2	63.7-87.1
PC ae C40:1	86.3	75.1-94.5	C2	75.6	63.7-86.2
PC aa C36:5	86.0	75.1-94.6	LysoPC a C17:0	74.8	61.4-86.3
PC aa C42:6	85.8	72.2-95.7	C10:1	72.7	59.2-83.2
C2/C0	85.6	75.8-94.5	SM C22:3	72.3	60.4-84.3
(C2+C3)/C0	85.3	74.1-93.9	LysoPC a C18:0	71.8	60.3-83.3
PC ae C38:0	85.2	74.0-94.5	C14:2	71.7	59.4-83.3

AA vs. CRC	AUC (%)	C.I. (95%)
PC aa C36:5	83.1	70.5-93.5
SM C24:0	80.4	65.0-91.5
PC ae C38:0	80.0	64.7-92.0
PC aa C36:6	79.8	66.9-91.8
PC aa C42:6	78.4	66.0-89.4
PC aa C34:4	78.1	63.4-90.7
PC aa C38:5	77.0	62.2-89.6
Total PC aa	77.0	62.4-89.0
Orn/Arg	76.8	61.3-90.0
Total PC	76.6	61.6-88.2

AC, acylcarnitine; lysoPC, monoacyl-glycerophosphocholine; PC aa, diacyl-glycerophosphocholine; PC ae, alkyl-acyl-glycerophosphocholine; SM, sphingomyelin.

Table S6. List of metabolites of the metabolomics analysis of both validation cohorts.

	Validation CRC			Validation AA		
(µM)	Ctrl (n= 29)	CRC (n=48)	P-value	Ctrl (n= 28)	AA (n=48)	P-value
**Acylcarnitines**						
C0	42 ± 9	44 ± 11	0.427	45 ± 9	44 ± 9	0.547
C2	8.35 ± 3.87	9.43 ± 2.95	0.172	7.86 ± 3.28	8.54 ± 2.70	0.063
C3	0.36 ± 0.09	0.38 ± 0.14	0.975	0.43 ± 0.13	0.41 ± 0.14	0.426
C3-DC (C4-OH)	0.04 ± 0.02	0.06 ± 0.03	**0.012**	0.04 ± 0.05	0.04 ± 0.03	0.387
C4	0.19 ± 0.06	0.21 ± 0.09	0.542	0.20 ± 0.07	0.23 ± 0.16	0.419
C5	0.13 ± 0.04	0.14 ± 0.07	0.821	0.15 ± 0.04	0.14 ± 0.04	0.267
C6 (C4:1-DC)	0.07 ± 0.03	0.08 ± 0.03	0.697	0.07 ± 0.04	0.08 ± 0.03	0.245
C7-DC	0.035 ± 0.021	0.048 ± 0.023	**0.004**	0.040 ± 0.021	0.038 ± 0.022	0.948
C8	0.20 ± 0.09	0.22 ± 0.11	0.690	0.21 ± 0.13	0.24 ± 0.11	0.071
C9	0.05 ± 0.02	0.04 ± 0.02	0.220	0.05 ± 0.02	0.05 ± 0.02	0.628
C10	0.31 ± 0.13	0.35 ± 0.16	0.403	0.32 ± 0.20	0.36 ± 0.17	0.085
C10:1	0.14 ± 0.07	0.15 ± 0.07	0.662	0.16 ± 0.08	0.15 ± 0.09	0.490

C10:2	<LOQ	<LOQ	-	<LOQ	<LOQ	-
C12	0.11 ± 0.07	0.14 ± 0.05	**0.031**	0.12 ± 0.08	0.12 ± 0.07	0.812
C12:1	0.09 ± 0.08	0.13 ± 0.07	**0.022**	0.11 ± 0.08	0.11 ± 0.07	0.607
C14	0.04 ± 0.02	0.05 ± 0.01	0.101	0.04 ± 0.02	0.04 ± 0.01	0.932
C14:1	0.11 ± 0.05	0.14 ± 0.05	**0.018**	0.11 ± 0.05	0.11 ± 0.04	0.630
C14:1-OH	0.013 ± 0.010	0.016 ± 0.009	0.192	0.02 ± 0.01	0.02 ± 0.01	0.742
C14:2	0.03 ± 0.02	0.04 ± 0.02	0.131	0.03 ± 0.02	0.04 ± 0.01	0.528
C16	0.12 ± 0.03	0.14 ± 0.03	**0.032**	0.13 ± 0.04	0.13 ± 0.03	0.923
C16:1	0.029 ± 0.027	0.048 ± 0.023	**0.005**	<LOQ	<LOQ	-
C16:1-OH	0.010 ± 0.005	0.011 ± 0.005	0.174	0.009 ± 0.007	0.009 ± 0.006	0.550
C16:2	0.005 ± 0.005	0.009 ± 0.006	**0.029**	<LOQ	<LOQ	-
C18	0.06 ± 0.01	0.06 ± 0.01	0.440	0.07 ± 0.02	0.06 ± 0.01	0.178
C18:1	0.15 ± 0.05	0.18 ± 0.04	**0.013**	0.15 ± 0.07	0.15 ± 0.04	0.299
C18:2	0.046 ± 0.017	0.052 ± 0.017	0.136	0.051 ± 0.018	0.048 ± 0.012	0.991
**Amino acids**						
Ala	442 ± 77	431 ± 90	0.592	339 ± 65	346 ± 82	0.914
Arg	134 ± 27	134 ± 28	0.939	127 ± 24	125 ± 28	0.714
Asn	45 ± 9	43 ± 9	0.269	36 ± 8	38 ± 7	0.310
Asp	26 ± 12	24 ± 11	0.328	25 ± 6.	28 ± 8	**0.035**
Cit	38 ± 12	37 ± 11	0.603	28 ± 10	33 ± 8	**0.015**
Gln	624 ± 109	607 ± 103	0.507	666 ± 99	710 ± 111	0.088
Glu	105 ± 52	99 ± 45	0.781	102 ± 33	102 ± 59	0.594
Gly	306 ± 63	276 ± 77	**0.028**	249 ± 52	288 ± 68	**0.014**
His	92 ± 12	86 ± 12	**0.034**	91 ± 13	89 ± 10	0.657
Ile	66 ± 18	81 ± 23	**0.003**	82 ± 22	81 ± 18	0.803
Leu	150 ± 44	164 ± 39	0.156	166 ± 37	167 ± 33	0.877
Lys	213 ± 33	205 ± 32	0.272	197 ± 27	196 ± 32	0.907
Met	21 ± 5	22 ± 4	0.358	24 ± 9	23± 6	0.689
Orn	89 ± 25	88 ± 24	0.748	81 ± 18	89 ± 20	0.073
Phe	74 ± 17	75 ± 12	0.854	80 ± 14	81 ± 14	0.778
Pro	145 ± 30	155 ± 39	0.424	198 ± 51	191 ± 51	0.527
Ser	157 ± 30	149 ± 27	0.261	136 ± 23	150 ± 20	**0.007**
Thr	116 ± 26	114 ± 22	0.810	99 ± 22	109 ± 22	0.063
Trp	62 ± 10	59 ± 9	0.152	63 ± 13	61 ± 14	0.596
Tyr	70 ± 17	73 ± 17	0.446	80 ± 23	75 ± 20	0.240
Val	207 ± 45	217 ± 38	0.262	247 ± 41	244 ± 36	0.715
**Biogenic amines**						
AcOrn	1.12 ± 0.60	1.51 ± 0.99	0.219	0.97 ± 0.53	1.10 ± 0.68	0.407
ADMA	0.55 ± 0.11	0.57 ± 0.15	0.748	0.65 ± 0.09	0.67 ± 0.12	0.291
Alpha AAA	0.69 ± 0.28	0.74 ± 0.27	0.233	0.19 ± 0.37	0.14 ± 0.30	0.626
Creatinine	72 ± 16	81 ± 19	**0.038**	84 ± 20	78 ± 20	0.193
Kynurenine	2.93 ± 0.85	3.04 ± 0.86	0.528	3.97 ± 0.88	3.71 ± 1.15	0.306
MetSO	1.99 ± 2.31	1.86 ± 2.35	0.574	2.17 ± 2.50	1.24 ± 2.03	**0.006**
Putrescine	0.13 ± 0.09	0.13 ± 0.07	0.846	0.17 ± 0.05	0.16 ± 0.06	0.386
Serotonin	0.65 ± 0.36	0.80 ± 0.51	0.274	0.69 ± 0.32	0.68 ± 0.45	0.659
Spermidine	0.13 ± 0.06	0.15 ± 0.05	0.168	0.18 ± 0.06	0.19 ± 0.04	0.261

Spermine	0.16 ± 0.04	0.16 ± 0.03	0.176	0.20 ± 0.14	0.19 ± 0.14	0.783
Taurine	107 ± 37	98 ± 35	0.278	77 ± 39	89 ± 35	0.059
Total DMA	1.29 ± 0.19	1.40 ± 0.31	0.131	0.87 ± 0.25	0.90 ± 0.20	0.369
OH-Pro	<LOQ	<LOQ	-	<LOQ	<LOQ	-
Sarcosine	4.31 ± 1.00	4.46 ± 1.32	0.871	4.30 ± 1.18	4.75 ± 1.06	0.096
**Glycerophospholipids**						
LysoPC a C16:0	152 ± 28	145 ± 38	0.305	138 ± 37	143 ± 35	0.576
LysoPC a C16:1	4.15 ± 1.13	3.97 ± 1.72	0.374	3.89 ± 1.29	4.12 ± 1.42	0.489
LysoPC a C17:0	3.01 ± 0.76	2.31 ± 0.77	**<0.001**	2.31 ± 0.89	2.60 ± 0.70	0.120
LysoPC a C18:0	40 ± 8	34 ± 11	**0.007**	34 ± 10	37 ± 9	0.243
LysoPC a C18:1	26 ± 5	25 ± 8	0.087	24 ± 8	25 ± 8	0.543
LysoPC a C18:2	33 ± 9	28 ± 10	**0.016**	31 ± 11	30 ± 11	0.636
LysoPC a C20:3	2.57 ± 0.72	2.22 ± 0.81	0.058	2.32 ± 0.70	2.41 ± 0.74	0.602
LysoPC a C20:4	8.40 ± 2.12	8.09 ± 2.92	0.270	8.12 ± 3.19	8.79 ± 2.62	0.151
LysoPC a C24:0	0.22 ± 0.01	0.17 ± 0.10	**<0.001**	0.22 ± 0.13	0.19 ± 0.06	0.779
LysoPC a C26:0	0.41 ± 0.33	0.34 ± 0.33	**0.002**	0.45 ± 0.37	0.33 ± 0.17	0.532
LysoPC a C26:1	0.14 ± 0.09	0.11 ± 0.08	**0.011**	0.16 ± 0.10	0.14 ± 0.06	0.504
LysoPC a C28:0	0.38 ± 0.27	0.24 ± 0.33	**0.002**	0.38 ± 0.32	0.31 ± 0.20	0.284
LysoPC a C28:1	0.65 ± 0.27	0.48 ± 0.24	**0.001**	0.56 ± 0.25	0.54 ± 0.19	0.872
PC aa C24:0	0.13 ± 0.09	0.08 ± 0.09	**0.002**	0.14 ± 0.12	0.10 ± 0.07	0.389
PC aa C28:1	3.50 ± 0.96	2.95 ± 0.75	**0.012**	3.00 ± 1.00	3.16 ± 0.71	0.463
PC aa C30:0	4.81 ± 1.83	3.93 ± 1.21	**0.036**	3.99 ± 1.57	4.51± 1.52	0.162
PC aa C32:0	13.54 ± 2.70	13.34 ± 2.46	0.736	12.27 ± 4.67	12.89 ± 2.98	0.136
PC aa C32:1	14.22 ± 6.71	15.11 ± 8.22	0.475	14.03 ± 6.91	14.37± 6.23	0.590
PC aa C32:2	4.21 ± 2.07	2.76 ± 1.32	**0.002**	3.06 ± 1.55	3.41 ± 1.31	0.142
PC aa C32:3	0.44 ± 0.14	0.33 ± 0.09	**<0.001**	0.31 ± 0.10	0.37 ± 0.10	**0.004**
PC aa C34:1	181 ± 31	191 ± 39	0.271	174 ± 40	184 ± 38	0.304
PC aa C34:2	301 ± 49	283 ± 46	0.098	279 ± 65	290 ± 50	0.410
PC aa C34:3	16.32 ± 4.84	13.85 ± 4.71	**0.030**	12.82 ± 4.24	14.78 ± 4.07	0.050
PC aa C34:4	1.80 ± 0.92	1.28 ± 0.58	**0.009**	1.39 ± 0.54	1.69 ± 0.60	**0.021**
PC aa C36:0	3.84 ± 1.37	3.38 ± 1.34	0.064	3.59 ± 1.31	3.56 ± 0.93	0.821
PC aa C36:1	39.09 ± 8.07	37.01 ± 12.67	0.123	35.14 ± 10.22	37.92 ± 10.20	0.256
PC aa C36:2	207 ± 33	182 ± 43	**0.008**	188 ± 47	196 ± 39	0.402
PC aa C36:3	114 ± 20	106 ± 27	0.202	102 ± 22	108 ± 23	0.250
PC aa C36:4	180 ± 39	181 ± 38	0.875	170 ± 46	188 ± 37	**0.042**
PC aa C36:5	22.17 ± 8.80	22.41 ± 10.51	0.709	20.17 ± 8.63	25.42 ± 12.30	0.106
PC aa C36:6	1.04 ± 0.58	0.75 ± 0.33	**0.027**	0.82 ± 0.37	0.97 ± 0.37	0.054
PC aa C38:0	3.05 ± 0.78	2.70 ± 0.91	**0.030**	2.72 ± 0.91	3.02 ± 0.68	0.109
PC aa C38:1	1.76 ± 0.79	1.50 ± 0.99	**0.008**	1.62 ± 0.84	1.48 ± 0.54	0.767
PC aa C38:3	52 ± 14	46 ± 13	0.073	44 ± 10	49 ± 12	0.073
PC aa C38:4	102 ± 29	96 ± 22	0.255	92 ± 24	104 ± 21	**0.018**
PC aa C38:5	44 ± 11	43 ± 10	0.500	38 ± 9	46 ± 11	**0.002**
PC aa C38:6	73 ± 19	70 ± 18	0.504	60 ± 18	76 ± 21	**0.001**
PC aa C40:1	0.50 ± 0.34	0.42 ± 0.36	0.116	0.42 ± 0.45	0.30 ± 0.36	0.166
PC aa C40:2	<LOQ	<LOQ	-	<LOQ	<LOQ	-
PC aa C40:3	1.05 ± 0.65	0.94 ± 0.66	0.227	1.13 ± 0.72	0.93 ± 0.51	0.311

PC aa C40:4	3.66 ± 1.06	3.41 ± 1.02	0.326	3.61 ± 1.19	3.59 ± 1.04	0.659
PC aa C40:5	11.61 ± 3.33	10.73 ± 2.87	0.291	9.98 ± 2.68	11.78 ± 3.49	**0.027**
PC aa C40:6	25 ± 8	23 ± 7	0.235	20 ± 6	26 ± 8	**0.003**
PC aa C42:0	0.66 ± 0.21	0.58 ± 0.20	0.070	0.60 ± 0.22	0.63 ± 0.18	0.349
PC aa C42:1	0.33 ± 0.14	0.29 ± 0.13	0.154	0.32 ± 0.15	0.30 ± 0.11	0.829
PC aa C42:2	0.33 ± 0.19	0.28 ± 0.17	0.090	0.35 ± 0.22	0.29 ± 0.14	0.260
PC aa C42:4	0.35 ± 0.26	0.29 ± 0.24	0.107	0.37 ± 0.24	0.29 ± 0.19	0.306
PC aa C42:5	0.46 ± 0.15	0.41 ± 0.16	**0.040**	0.45 ± 0.16	0.45 ± 0.15	0.893
PC aa C42:6	0.56 ± 0.16	0.46 ± 0.16	**0.004**	0.50 ± 0.15	0.53 ± 0.14	0.146
PC ae C30:0	0.53 ± 0.15	0.44 ± 0.14	**0.010**	0.48 ± 0.21	0.49 ± 0.13	0.712
PC ae C30:1	<LOQ	<LOQ	-	<LOQ	<LOQ	-
PC ae C30:2	0.17 ± 0.09	0.14 ± 0.08	**0.004**	0.16 ± 0.09	0.14 ± 0.06	0.628
PC ae C32:1	2.67 ± 0.58	2.48 ± 0.47	0.115	2.47 ± 1.55	2.52 ± 0.53	0.840
PC ae C32:2	0.65 ± 0.16	0.56 ± 0.12	**0.017**	0.58 ± 0.32	0.60 ± 0.12	0.736
PC ae C34:0	1.63 ± 0.44	1.33 ± 0.32	**0.005**	1.27 ± 0.40	1.45 ± 0.38	0.057
PC ae C34:1	8.72 ± 1.75	7.84 ± 1.69	**0.032**	6.97 ± 2.11	7.90 ± 1.65	**0.035**
PC ae C34:2	9.13± 1.72	7.68 ± 2.46	**0.003**	7.15 ± 2.39	8.27 ± 2.03	**0.033**
PC ae C34:3	7.40 ± 2.14	6.14 ± 1.91	**0.009**	6.19 ± 2.22	6.65 ± 1.86	0.643
PC ae C36:0	1.05 ± 0.27	1.04 ± 0.28	0.805	1.01 ± 0.32	1.00 ± 0.23	0.651
PC ae C36:1	8.89 ± 4.84	7.11 ± 3.70	**0.024**	7.58 ± 3.79	7.06 ± 2.86	0.970
PC ae C36:2	14.60 ± 3.44	11.52 ± 3.51	**<0.001**	11.01 ± 3.26	12.19 ± 3.32	0.142
PC ae C36:3	6.56 ± 1.14	5.66 ± 1.85	**0.010**	5.52 ± 1.60	6.14 ± 1.49	0.091
PC ae C36:4	16.86 ± 3.75	16.57 ± 4.41	0.765	16.13 ± 4.30	18.22 ± 3.98	**0.035**
PC ae C36:5	10.44 ± 2.74	10.17 ± 2.50	0.659	10.28 ± 2.93	11.47 ± 2.55	0.067
PC ae C38:0	2.00 ± 0.70	1.64 ± 0.50	**0.026**	1.67 ± 0.60	1.94 ± 0.56	**0.018**
PC ae C38:1	<LOQ	<LOQ	-	<LOQ	<LOQ	-
PC ae C38:2	<LOQ	<LOQ	-	<LOQ	<LOQ	-
PC ae C38:3	7.27 ± 4.80	5.79 ± 3.78	**0.037**	6.95 ± 4.33	5.76 ± 3.51	0.454
PC ae C38:4	13.51 ± 2.96	12.03 ± 2.07	**0.011**	11.87 ± 2.45	13.08± 2.38	**0.037**
PC ae C38:5	17.11 ± 3.45	16.79 ± 3.80	0.719	16.07 ± 3.99	18.27 ± 3.43	**0.013**
PC ae C38:6	6.82 ± 1.70	6.29 ± 1.78	0.205	5.92 ± 1.53	7.19 ± 1.60	**0.001**
PC ae C40:1	1.56 ± 0.63	1.28 ± 0.57	**0.021**	1.56 ± 0.74	1.46 ± 0.50	0.996
PC ae C40:2	2.72 ± 1.10	2.43 ± 1.08	0.117	2.38± 1.04	2.35 ± 0.81	0.702
PC ae C40:3	2.38 ± 2.22	1.90 ± 1.93	**0.044**	2.54 ± 2.04	1.85 ± 1.81	0.491
PC ae C40:4	3.84 ± 1.80	3.25 ± 1.42	0.069	3.76 ± 1.64	3.36 ± 1.37	0.322
PC ae C40:5	4.34 ± 1.96	3.70 ± 1.33	0.112	4.09 ± 1.62	3.90 ± 1.26	0.617
PC ae C40:6	5.28 ± 1.27	4.54 ± 1.21	**0.009**	4.23 ± 1.02	5.05 ± 1.00	**0.001**
PC ae C42:1	0.64 ± 0.39	0.54 ± 0.41	0.057	0.69 ± 0.47	0.55 ± 0.35	0.369
PC ae C42:2	0.77 ± 0.35	0.66 ± 0.32	0.114	0.76 ± 0.39	0.69 ± 0.27	0.598
PC ae C42:3	1.06 ± 0.49	0.90 ± 0.45	0.081	1.05 ± 0.52	0.95 ± 0.36	0.441
PC ae C42:4	1.10 ± 0.38	0.97 ± 0.33	0.074	1.05 ± 0.36	1.00 ± 0.33	0.494
PC ae C42:5	2.77 ± 0.85	2.53 ± 0.62	0.247	2.62 ± 0.76	2.63 ± 0.63	0.851
PC ae C44:3	0.21 ± 0.14	0.19 ± 0.15	0.089	0.22 ± 0.15	0.19 ± 0.11	0.730
PC ae C44:4	0.51 ± 0.15	0.47 ± 0.12	0.207	0.46 ± 0.13	0.47 ± 0.12	0.464
PC ae C44:5	1.94 ± 0.62	1.89 ± 0.45	0.866	1.82 ± 0.72	2.00 ± 0.48	0.203
PC ae C44:6	1.42 ± 0.49	1.29 ± 0.34	0.326	1.26 ± 0.39	1.37 ± 0.33	0.170

**Sphingomyelins**						
SM (OH) C14:1	9.59 ± 2.20	8.07 ± 2.53	**0.003**	7.22 ± 2.26	8.04 ± 1.77	0.081
SM (OH) C16:1	5.05 ± 0.89	4.31 ± 1.21	**0.005**	3.70 ± 1.07	4.37 ± 0.89	**0.004**
SM (OH) C22:1	19.27 ± 4.18	16.12 ± 4.60	**0.001**	14.20 ± 3.43	16.71 ± 3.61	**0.004**
SM (OH) C22:2	19.10 ± 3.48	15.50 ± 4.85	**0.001**	13.51 ± 3.54	16.33 ± 3.51	**0.001**
SM (OH) C24:1	1.85 ± 0.41	1.64 ± 0.46	**0.044**	1.45 ± 0.35	1.71 ± 0.37	**0.003**
SM C16:0	143 ± 22	138 ± 22	0.345	122 ± 25	134 ± 19	**0.025**
SM C16:1	25.10 ± 4.60	22.69 ± 4.56	**0.028**	20.56 ± 4.72	23.08 ± 4.08	**0.017**
SM C18:0	33.75 ± 5.17	32.23 ± 5.85	0.252	27.21 ± 6.93	31.65 ± 5.03	**0.005**
SM C18:1	15.80 ± 2.82	14.11 ± 3.23	**0.018**	12.19 ± 3.37	14.72 ± 3.04	**0.001**
SM C20:2	0.89 ± 0.19	0.81± 0.16	0.066	0.68 ± 0.19	0.76 ± 0.12	0.054
SM C22:3	1.78 ± 0.46	2.04 ± 0.56	**0.037**	1.34 ± 0.44	1.45 ± 0.38	0.539
SM C24:0	32.29 ± 7.22	30.29 ± 6.80	0.310	27.36 ± 5.28	29.84 ± 5.44	0.057
SM C24:1	63.39 ± 11.56	66.52 ± 10.64	0.229	55.63 ± 12.91	64.34 ± 10.72	**0.002**
SM C26:0	0.30 ± 0.07	0.27 ± 0.09	0.081	0.25 ± 0.08	0.29 ± 0.07	**0.036**
SM C26:1	0.60 ± 0.12	0.61 ± 0.16	0.908	0.53 ± 0.17	0.61 ± 0.14	**0.028**
**Monosaccharides**						
H1	5798 ± 1731	6070 ± 1932	0.418	5481 ± 1280	5636 ± 1495	0.698
**Ratios and sums**						
**Acylcarnitines**						
C2/C0	0.203 ± 0.92	0.231 ± 0.100	0.211	0.180 ± 0.090	0.201 ± 0.074	0.085
(C2+C3)/C0	0.212 ± 0.92	0.239 ± 0.100	0.223	0.190 ± 0.090	0.211 ± 0.074	0.079
CPT-I-ratio	0.011 ± 0.04	0.013 ± 0.006	0.138	0.010 ± 0.005	0.010 ± 0.003	0.338
Total AC/C0	0.262 ± 0.108	0.298 ± 0.125	0.192	0.238 ± 0.106	0.263 ± 0.086	0.085
Total AC-DC/Total AC	0.0034 ± 0.0011	0.0040 ± 0.0013	**0.029**	0.0033 ± 0.0018	0.0035 ± 0.0015	0.627
Total AC-OH/Total AC	0.0018 ± 0.0007	0.0022 ± 0.0010	**0.027**	0.0016 ± 0.0014	0.0016 ± 0.0011	0.838
**Amino acids and biogenic amines**						
ADMA/Arg	0.0042 ± 0.0011	0.0043 ± 0.0012	0.674	0.005 ± 0.001	0.006 ± 0.001	0.248
Aromatic AA	206 ± 40	206 ± 32	0.966	222 ± 40	217 ± 41	0.568
BCAA	423 ± 102	462 ± 93	0.089	495 ± 93	492 ± 81	0.876
Calc. SDMA/Arg	0.006 ± 0.002	0.006 ± 0.002	0.263	0.0019 ± 0.0018	0.0018 ± 0.0015	0.872
Cit/Arg	0.289 ± 0.093	0.281 ± 0.098	0.494	0.220 ± 0.058	0.273 ± 0.068	**0.001**
Cit/Orn	0.443 ± 0.155	0.434 ± 0.142	0.795	0.355 ± 0.121	0.380 ± 0.082	0.278
Essential AA	1001 ± 166	1022 ± 131	0.531	1049 ± 149	1052 ± 145	0.928
Fischer ratio	2.063 ± 0.388	2.249 ± 0.381	**0.042**	2.263 ± 0.391	2.308 ± 0.366	0.804
Glucogenic AA	905 ± 105	857 ± 139	0.115	725 ± 113	784 ± 139	0.062
Kyn/Trp	0.047 ± 0.012	0.052 ± 0.013	0.117	0.065 ± 0.016	0.061 ± 0.015	0.249
MetSO/Met	0.128 ± 0.191	0.104 ± 0.155	0.418	0.128 ± 0.197	0.073 ± 0.144	**0.007**
Non-essential AA	2053 ± 182	1992 ± 247	0.247	1958 ± 252	2052 ± 275	0.141
Orn/Arg	0.704 ± 0.285	0.681 ± 0.220	0.916	0.648 ± 0.151	0.737 ± 0.199	0.055
Putrescine/Orn	0.0015 ± 0.0010	0.0015 ± 0.0009	0.528	0.0022 ± 0.0007	0.0019 ± 0.0008	0.058

Ser/Trp	0.011 ± 0.008	0.014 ± 0.009	0.101	0.0112 ± 0.0055	0.0116 ± 0.0073	0.940
Spermidine/Putrescine	1.28 ± 0.80	1.76 ± 1.59	0.258	1.07 ± 0.28	1.27 ± 0.37	**0.023**
Spermine/Spermidine	1.32 ± 0.36	1.24 ± 0.41	0.296	1.11 ± 0.87	0.95 ± 0.74	0.376
Total AA	3181 ± 338	3139 ± 341	0.596	3116 ± 381	3227 ± 411	0.247
Total DMA/Arg	0.010 ± 0.003	0.011 ± 0.003	0.248	0.007 ± 0.002	0.007 ± 0.002	0.286
Tyr/Phe	0.943 ± 0.155	0.975 ± 0.200	0.474	0.991 ± 0.192	0.928 ± 0.191	0.170
**Lipids**						
MUFA PC	263 ± 50	266 ± 62	0.983	248 ± 58	260 ± 55	0.370
MUFA PC/SFA PC	8.50 ± 1.14	9.37 ± 1.36	**0.005**	9.17 ± 1.80	8.82 ± 0.92	0.755
PUFA PC	1304 ± 202	1213 ± 200	0.059	1175 ± 206	1284 ± 207	**0.028**
PUFA PC/MUFA PC	5.04 ± 0.79	4.64 ± 0.56	**0.011**	4.84 ± 0.71	5.01 ± 0.50	0.230
PUFA PC/SFA PC	42.60 ± 7.00	43.15 ± 6.08	0.720	43.95 ± 7.82	44.01 ± 4.89	0.970
SFA PC	31 ± 7	29 ± 6	0.091	27.9 ± 8.8	29.7 ± 6.5	0.328
Total lysoPC	271 ± 46	249 ± 67	0.117	246 ± 66	254 ± 62	0.564
Total lysoPC/total PC	0.17 ± 0.03	0.17 ± 0.04	0.333	0.17 ± 0.04	0.16 ± 0.03	0.333
Total PC	1601 ± 244	1511 ± 256	0.134	1453 ± 260	1576 ± 263	0.051
Total PC aa	1430 ± 219	1360 ± 236	0.201	1300 ± 240	1415 ± 245	0.051
Total PC ae	171 ± 36	151 ± 31	**0.012**	153 ± 35	161 ± 29	0.252
Total (PC+SM)	2243 ± 315	2113 ± 329	0.091	2007 ± 346	2179 ± 333	**0.036**
Total SM	371 ± 55	353 ± 58	0.173	308 ± 61	348 ± 49	**0.003**
Total SM (non-OH)	317 ± 46	307 ± 47	0.405	268 ± 53	301 ± 42	**0.004**
Total SM (OH)	54.9 ± 10.4	45.6 ± 13.0	**<0.001**	40.08 ± 10.05	47.17 ± 9.52	**0.003**
Total SM (OH)/ total SM (non-OH)	0.17 ± 0.02	0.15 ± 0.03	**<0.001**	0.150 ± 0.025	0.157 ± 0.023	0.232
Total SM/total PC	0.23 ± 0.03	0.24 ± 0.04	0.607	0.213 ± 0.029	0.224 ± 0.029	0.138
Total SM/total PL pool	0.17 ± 0.01	0.17 ± 0.02	0.526	0.154 ± 0.017	0.161 ± 0.019	0.085

AA, amino acid; AC, acylcarnitine; AC-DC, dicarboxyacylcarnitine; AC-OH, hydroxylated acylcarnitine; Arg, arginine; BCAA, branched chain amino acid; Cit, citrulline; DMA, dimethylarginine; Kyn, kynurenine; lysoPC, monoacyl-glycerophosphocholine; Met, methionine; MetSO, methionine sulfoxide; MUFA; mono-unsaturated fatty acid; Orn, ornithine; PC aa, diacyl-glycerophosphocholine; PC ae, alkyl-acyl-glycerophosphocholine; Phe, phenylalanine; PL, phospholipid; PUFA, polyunsaturated fatty acid; SFA, saturated fatty acid; Ser, serine; SM, sphingomyelin; Trp, tryptophan; Tyr, tyrosine; data are expressed in µM as means ± standard deviation; P-values are assessed by ANOVA (Benjamini-Hochberg or Tamhane post hoc analysis) or Kruskal-Wallis test; P-values <0.05 were considered to indicate statistical significance and are marked in bold; metabolites are sorted in alphabetical order.

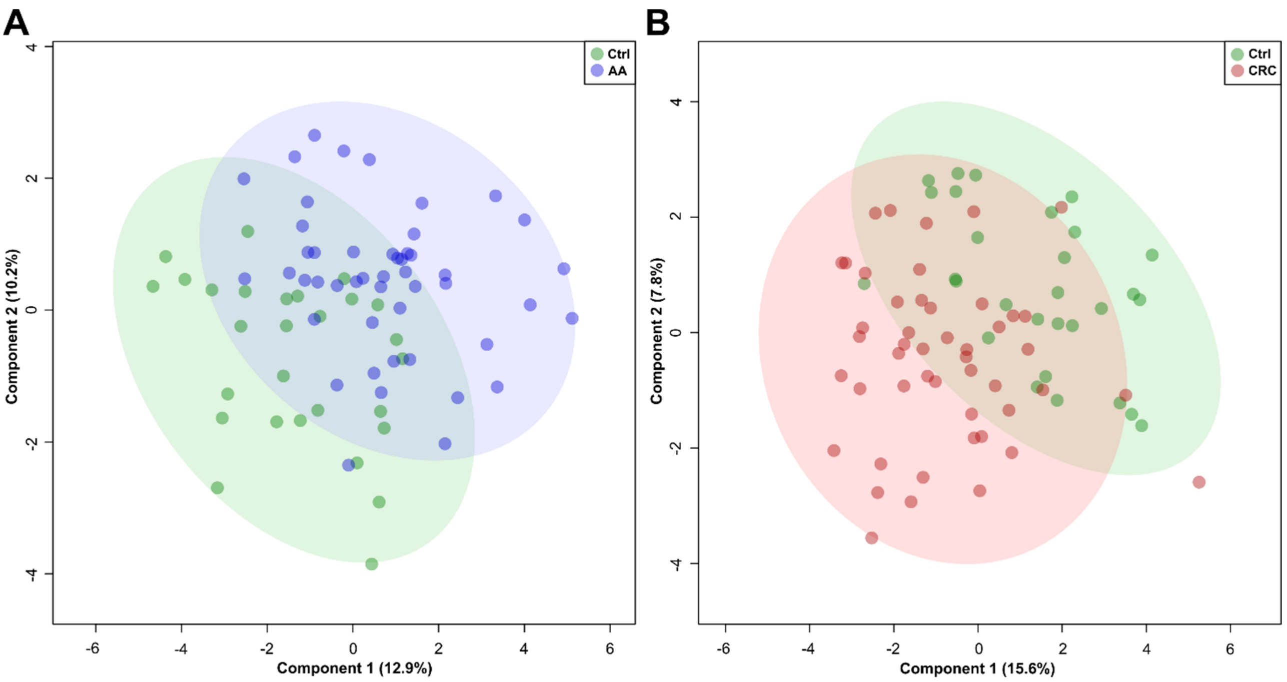

Figure S1. Sparse partial least discriminant analysis (sPLS-DA) of the two validation cohorts. (**A**) validation AA; (**B**) validation CRC; circles indicate 95% CI; adapted from MetaboAnalyst 4.0.

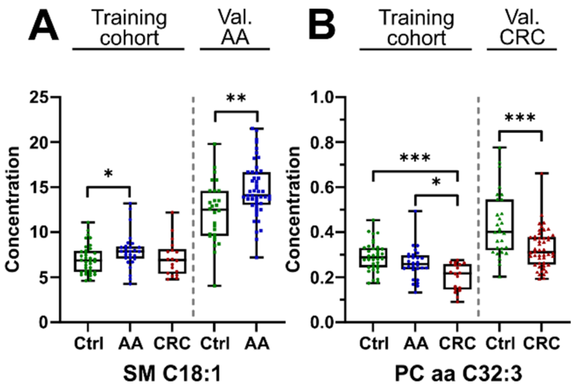

Figure S2. Selected validated metabolites of the training cohort and the validation cohorts. (**A**) SM C18:1 as one validated metabolite of the training cohort and validation AA; (**B**) PC aa C32:3 as one validated metabolite of the training cohort and validation CRC; data are expressed in μM; * indicates P-values <0.05, ** P-values <0.01, *** P-values <0.001.
